# LMCT‐Driven Iron Photocatalysis: Mechanistic Insights and Synthetic Applications

**DOI:** 10.1002/chem.202502185

**Published:** 2025-09-10

**Authors:** Amrita Chaudhuri, Luca Mareen Denkler, Qing Zhuo, Anup Mandal, Ala Bunescu

**Affiliations:** ^1^ Kekulé Institute of Organic Chemistry and Biochemistry University of Bonn Gerhard‐Domagk‐Straße 1 53121 Bonn Germany

**Keywords:** C─C and C─heteroatom bond formation, C─H functionalization, hydrogen atom transfer (Hat), iron photocatalysis, ligand‐to‐metal charge transfer (LMCT), single‐electron transfer (SET)

## Abstract

Iron‐based photocatalysis has emerged as a sustainable and versatile platform for facilitating a wide range of chemical transformations, offering an appealing alternative to precious metal photocatalysts. Among the various activation modes, ligand‐to‐metal charge transfer (LMCT)‐driven homolysis of Fe(III)–L(ligand) bonds has garnered considerable attention due to its ability to generate reactive radical species under mild conditions, without requiring the matching of substrates’ redox potentials. In this review, we present a comprehensive overview of recent developments in LMCT‐driven iron photocatalysis, with a particular focus on both mechanistic insights and synthetic applications published in the last five years. We classify Fe(III)–L homolysis into four major categories based on the nature of the coordinated ligand: halides, carboxylates, alkoxides, and azide. For a few cases, mechanistic understanding derived from spectroscopic studies, computational modeling, and kinetic investigations is discussed in more detail. We further highlight the expanding repertoire of synthetic transformations enabled by LMCT‐driven iron photocatalysis, including C─H functionalization, alkene functionalization, cross‐coupling, oxidation, and radical‐mediated bond formation. Finally, we provide future perspectives on the continued development of LMCT‐based iron photocatalysis as a broadly applicable platform for sustainable organic synthesis. This review aims to serve as a valuable resource for researchers interested in leveraging the full potential of LMCT‐mediated iron photocatalysis in modern organic chemistry.

## Graphical abstract

LMCT‐driven iron photocatalysis: This review discusses recent developments in ligand‐to‐metal charge transfer (LMCT)‐driven iron photocatalysis, emphasizing mechanistic insights and diverse synthetic applications. By classifying Fe(III)–L bond homolysis pathways, it highlights iron's potential as a sustainable alternative to precious metals for radical‐mediated reactions under mild conditions.

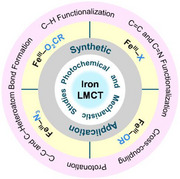

## 1 Introduction

Photocatalysis enables organic transformations to be powered by photon energy through single‐electron transfer (SET), allowing radical reactions to proceed with more environmentally friendly reagents, under milder conditions, thus advancing the sustainability goals of organic synthesis.^[^ ^1^ ^]^ Radical‐mediated organic transformations have witnessed a rapid resurgence thanks to the increased use of photoredox catalysis. Photoredox catalysts, which can mostly be either transition metal‐based complexes or organic dyes, absorb visible or UV light to generate highly reactive intermediates that undergo electron or charge transfer. These excited states are often highly oxidizing or reducing and relatively long‐lived, enabling productive interactions between the excited photocatalyst and the substrate, which ultimately drive the desired transformation forward.^[^ ^2^ ^]^ From a green chemistry perspective, organic photoredox systems provide an appealing alternative to metal‐based systems, which typically rely on precious metals such as iridium (Ir) and ruthenium (Ru), which are expensive and in limited supply. Despite the clear sustainability advantages of organophotoredox catalysis, metal‐based systems remain dominant, due to their broader redox windows, longer excited‐state lifetimes, and potential for dual catalytic roles.^[^ ^3^ ^]^ One of the key challenges in photoredox catalysis is matching the redox potentials of the catalyst and substrate to ensure efficient SET. Another critical factor is the excited‐state lifetime of the photocatalyst.^[^ ^4^ ^]^ Since SET is an intermolecular, outer‐sphere process, sufficient time must be allowed for the catalyst and substrate to diffuse through the solvent and form a productive encounter complex. For effective bimolecular quenching, the excited state lifetime should typically be in the order of the nanoseconds.^[^ ^5^ ^]^ For this reason, Ir‐based complexes, which have the excited‐state lifetimes on the order of several hundred to thousands of nanoseconds, are particularly well‐suited to serve as effective photoredox catalysts.^[^ ^6^ ^]^

**Scheme 1:**
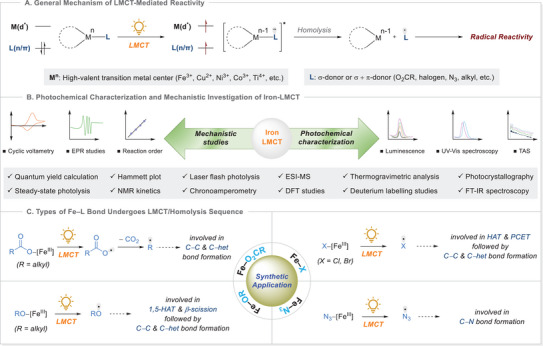
Scope and structure of review. EPR, electron paramagnetic resonance; TAS, transient absorption spectroscopy; NMR, nuclear magnetic resonance; ESI‐MS, electrospray ionization mass spectrometry; DFT, density functional theory; FT‐IR, Fourier‐transform infrared; het, heteroatom; hat, hydrogen atom transfer.

An alternative approach to generating open‐shell species from the excited states of metal complexes involves the ligand‐to‐metal charge transfer (LMCT) manifold that proceeds via inner sphere electron transfer (ISET). Compared to photoredox reactivity that proceeds via outer sphere electron transfer (OSET), ISET does not require precise redox potential tuning and high excited state lifetime, which makes LMCT applicable to a broader range of substrates beyond those accessible via traditional photoredox catalysis (Scheme 1A).^[^ ^3^, ^7^ ^]^ Since bond cleavage is facilitated by an intramolecular electronic reorganization, LMCT is often more efficient and selective in generating radicals than photoredox processes that rely on diffusion‐limited OSET mechanisms.^[^ ^8^ ^]^ Additionally, LMCT is common in earth‐abundant first‐row transition metals such as Fe, Mn, and Co, which offer cheaper, scalable, and more environmentally friendly alternatives to noble‐metal photoredox catalysis, overall establishing the substrate activation via LMCT as a more direct, efficient, and sustainable approach to radical generation, further enhancing its value as a tool for modern synthetic applications.^[^ ^9^ ^]^ Fe‐based LMCT processes are superior in photoredox catalysis due to iron's abundance and cost‐effectiveness compared to metals like Cu, Ni, Co, and Zr. Iron complexes exhibit a strong absorption in the visible and near‐UV regions, enabling fast charge transfer and efficient radical generation for organic transformations.^[^ ^10^ ^]^ With multiple accessible oxidation states, iron exhibits excellent redox versatility, making it ideal for C─C/C─heteroatom bond formation. Additionally, iron is nontoxic, making it safer and more user‐friendly. These properties make it the preferred choice for sustainable iron‐based LMCT processes.^[^ ^11^ ^]^

### 1.1 Scope of this Review

This review provides a comprehensive analysis of iron photocatalyzed LMCT‐mediated homolysis from 2020 to the present moment. We begin by discussing the fundamental photophysical and mechanistic principles governing LMCT activation in iron complexes (Scheme 1B). We then categorize Fe(III)–L homolysis into four major classes: Fe(III) with halide, carboxylate, alkoxy, and azide substrates and explore its key synthetic applications after forming active radical intermediate which includes C─C and C─heteroatom bond formation, hydrogen atom transfer (HAT), and cross‐coupling reactions, and others (Scheme 1C). Furthermore, we highlight recent advances in catalyst design and mechanistic insights that have expanded the role of iron in photocatalysis. Finally, we address the challenges and prospects of this field, underscoring the potential of iron‐based LMCT catalysis for sustainable and scalable chemical transformations. Most important reviews addressing LMCT for 3d metals were reported recently by Juliá,^[^ ^12^ ^]^ Jin,^[^ ^13^ ^]^ and Reiser.^[^ ^14^ ^]^ A more mechanistically oriented reviews were published by Dempsey,^[^ ^9^ ^]^ Fukuzumi,^[^ ^15^ ^]^ and König.^[^ ^16^ ^]^ In addition, there are reviews on iron photocatalysis by Browne,^[^ ^17^ ^]^ Wärnmark,^[^ ^18^ ^]^ and Zeng.^[^ ^19^ ^]^ The current review represents a complementary work to those published previously, with a particular highlight on iron‐photocatalyzed transformation via LMCT mode of action.

### 1.2 Historical Background of Iron Photochemistry Based on LMCT

The photoactivity of Fe(III) complexes has been recognized for decades, yet it remains relatively underexplored. The first photoinduced reduction of iron(III) oxalates was reported by Parker back in 1953.^[^ ^20^ ^]^ In this study, it was found that the ferrioxalate undergoes photoexcitation upon irradiation, releasing carbon dioxide as a byproduct through LMCT, in which an electron is transferred from the oxalate ligand to the Fe(III) center, generating a reduced Fe(II) species. Later, carboxylate ligands were also found to undergo decarboxylation via LMCT in Fe(III) complexes upon irradiation.^[^ ^21^ ^]^ The first application of the iron‐photoinduced LMCT principle to the activation of carboxylic acids in the context of organic synthesis was reported by Sugimori in 1986 for the alkylation of heteroarenes via Minisci‐type reaction.^[^ ^22^ ^]^ In this work the alkyl radical was generated from carboxylic acids using Fe_2_(SO_4_)_3_ as a stoichiometric photooxidant. First, the carboxylic acid coordinates to the Fe(III) center and then oxygen center radical is generated in a photoinduced LMCT/homolysis sequence. Subsequent decarboxylation leads to the formation of an alkyl radical, which serves as a key intermediate in the alkylation reaction. Despite the long‐known photoreactivity of Fe(III) complexes and this earlier application to organic synthesis, iron‐catalyzed LMCT platform remained underexplored until recently. In 2019, building on the pioneering work of Sugimori, Jin and coworkers successfully utilized a catalytic amount of iron(III)‐based photocatalyst for a Minisci‐type reaction.^[^ ^23^ ^]^ In this reaction, alkyl carboxylic acids generate alkyl radicals through photoinduced LMCT, enabling their coupling with heteroarenes. A crucial aspect of the process was the regeneration of Fe(III) from Fe(II), formed during the LMCT step, which was achieved using a persulfate‐type oxidant. This breakthrough demonstrated the potential of Fe(III) complexes as photocatalysts, significantly broadening their applicability in organic transformations. In recent years, several review articles have summarized transition metal‐photocatalyzed decarboxylative functionalization. The present article further contributes to this field by specifically highlighting recent progress in iron‐catalyzed LMCT‐mediated decarboxylative transformations.^[^ ^24^ ^]^

## 2 Photochemical Transformations via LMCT Excited States of Iron

### 2.1 Overview of Iron‐Photoinduced LMCT

Photocatalytic transformations have garnered significant attention for their ability to generate open‐shell species under mild conditions with high selectivity.^[^ ^17^, ^25^ ^]^ MLCT (metal to ligand charge transfer) is the most common in photoredox reactions due to its extended excited‐state lifetime and notable photochemical stability. However, the use of precious metals like ruthenium or iridium in MLCT processes is constrained by their redox potential windows and high costs, limiting their applicability.^[^ ^25^, ^26^ ^]^ In contrast, LMCT involves the metal center acting as the acceptor, with electrons being transferred from ligand‐based orbitals to metal‐based receptor orbitals, resulting in the oxidation of the ligand. This mechanism exhibits unique reactivity and offers an innovative approach for photochemical transformations using first‐row transition metals. There has been a notable increase in research dedicated to the application of iron as a photocatalyst, attributed to its nontoxic nature, ease of handling, and commendable photochemical properties. This section aims to discuss the iron photochemistry associated with LMCT‐type photoactivation.^[^ ^9^, ^12^, ^15^ ^]^

**Scheme 2:**
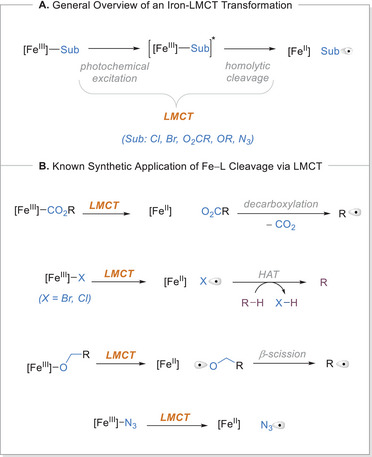
General overview and type of relativities associated with LMCT. Sub, substrate.

The LMCT reaction pathway typically entails electron transitions from the ligand to the vacant orbitals of the metal center of the excited iron‐ligand (Fe─L) complex (Scheme 2). Upon light excitation, this excited state may facilitate the visible light‐induced homolysis (VLIH) of the iron–L bond, producing a formally reduced metal center and oxidized ligand radical.^[^ ^9^, ^11^, ^25^, ^27^ ^]^ Iron(III) emerges as an excellent candidate for LMCT due to the presence of low‐lying orbitals that are not fully occupied. Notably, high‐spin Fe(III) complexes are particularly advantageous as they disfavor unproductive d‐d transitions. Electron donation during photoinduced charge transfer (PCT) can be facilitated by ligands those are strong/moderate σ‐ and/or π‐donors. Examples of such donors include halogens (Br, Cl), carboxylates (O_2_CR), alkoxides (OR), and azide (N_3_). The π‐donor characteristics of these ligands promote the formation of a relatively electron‐rich bond between the ligand and the metal center, significantly enhancing the efficiency of the LMCT process.^[^ ^9^, ^12^, ^15^ ^]^ In this section, we provide a concise overview of the most extensively studied systems, employing tangible examples to illustrate the discussed principles.

### 2.2 Formation of Alkyl Radicals Over Decarboxylative Transformations From Iron‐Photoinduced LMCT

**Scheme 3:**
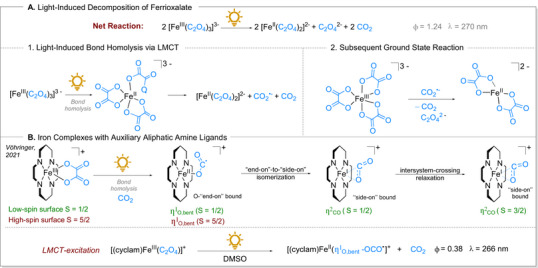
The formation of alkyl radicals during decarboxylative transformations over iron‐photoinduced LMCT. Cyclam, 1,4,8,11‐tetraazacyclotetradecane.

Potassium ferrioxalate (K_3_[Fe(C_2_O_4_)_3_]) is extensively utilized as a chemical actinometer for the measurement of photon fluxes and the determination of quantum yields in photochemical reactions (Scheme 3A).^[^ ^28^ ^]^ This compound is particularly advantageous owing to its heightened sensitivity to light and its well‐established photochemical properties. The foundational reaction for photon flux determination was initially documented by Allmand and Webb.^[^ ^29^ ^]^ Hatchard and Parker reported the use of potassium ferrioxalate as an actinometer and conducted thorough investigations into the quantum yields of ferrioxalate under different conditions.^[^ ^20^, ^30^ ^]^ The ferrioxalate anion Fe^III^(C_2_O_4_)_3_^3−^ remains stable in the dark but undergoes decomposition if subjected to light. Fe(III)‐oxalate complexes exhibit charge‐transfer (CT) bands with maxima at 260 nm, overlapping with a strong absorption band of the ligands at 210 nm.^[^ ^31^ ^]^ The rather weak ligand field from the oxalates results in a high‐spin (S,5/2) electron configuration at the metal.^[^ ^32^ ^]^ The LMCT excited state of ferrioxalate experiences photoreduction, whereby Fe(III) is reduced to Fe(II) and one oxalate ligand is oxidized to carbon dioxide (Fe^III^(C_2_O_4_)_3_^3−^ + hν → Fe^II ^+ 2 CO_2 _+ 3 C_2_O_4_^2−^).^[^ ^9^, ^33^ ^]^ This transformation has a quantum yield of Φ = 1.24 (270 nm). The underlying mechanism of the light induced decomposition was studied by several groups over pump/probe transient absorption spectroscopy. After the excited state decarboxylates the formed carbon dioxide radical anion (CO_2_^•−^) reduces another ferrioxalate to Fe(II). This means that more than one Fe^III^(C_2_O_4_)_3_^3−^ can be consumed with a single photochemical transformation.^[^ ^28^, ^34^ ^]^ The utilization of alternative dicarboxylate ligands, which also exhibit strong coordination to iron, results in the acquisition of a pronounced LMCT absorption band. Photolysis typically occurs via the mechanism depicted in Scheme 3.^[^ ^35^ ^]^

The photochemistry of Fe(III) oxalate complexes is highly dependent on the ligand environment. This was demonstrated by Vöhringer and coworkers by studying the photophysical properties of iron complexes bound to cyclic tetradentate auxiliary ligand (Scheme 3B). Using electron paramagnetic resonance (EPR), a high‐spin state iron (III) carboxylate [S = 5/2 (in DMSO)] is favored in solution. While in the neat powder, a low‐spin configuration (S = 1/2) is energetically favored bearing the cyclam ligand were determined. The complex was further investigated utilizing transient absorption to determine the fate of carbon dioxide after the photoexcitation. In DMSO solution, the dynamics of the photolysis start from the sextet ground‐state of [Fe ^III^(cyclam)(C_2_O_4_)]^+^ (S = 5/2) with some thermal population in the doublet excited state (S = 1/2). In the case of a high‐spin surface, after the loss of neutral carbon dioxide, the primary product relaxes to a stable high‐spin iron(II) species bound carbon dioxide radical anion in bent‐O‐“end‐on” fashion (η^1^_O,bent_, S = 5/2). In the case of a low‐spin surface, the release of neutral CO_2_ from the complex results in the formation of a low‐spin iron(II) complex with carbon dioxide anion that is also O‐end bounded to the metal complex (η^1^_O,bent_, S = 1/2). This low‐spin iron(II) complex subsequently undergoes “end‐on”‐to‐“side‐on” structural isomerization to furnish a low‐spin Fe(I) complex (η^2^_CO_, S = 1/2) bound to a neutral carbon dioxide. This low‐valent iron complex can further undergo intersystem‐crossing relaxation without significant structural changes to the intermediate‐spin ground state (η^2^_C__O_, S = 3/2). The 266 nm LMCT‐excitation of [Fe^III^(cyclam)(C_2_O_4_)]+ in DMSO delivers the Fe(II) bound carbon dioxide radical anion in bent‐O‐“end‐on” fashion with a primary photochemical quantum yield for CO_2_‐release of 38%. The ability to stabilize the carbon dioxide radical anion is attributed to the secondary ligand, which enables the tuning of spin states. This study clearly demonstrates the advantages of using supporting ligands to tune and control iron‐photoinduced LMCT reactivities.^[^ ^32^, ^36^ ^]^

**Scheme 4:**
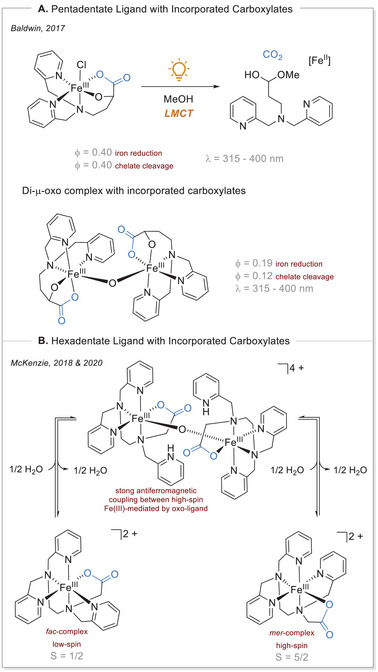
Influence of ligand and structure on the photochemistry of iron complexes.

Various research groups have incorporated carboxylates into multidentate ligands and studied these iron complexes in detail.^[^ ^17^, ^37^ ^]^ The work of the Baldwin group deserves special mention, as they developed a pentadentate ligand from an α‐hydroxy amino acid (Scheme 4). Homoleptic iron(III) complexes were synthesized and subjected to UVA (ultraviolet A) irradiation in methanol, thereby stimulating the LMCT, which in turn led to decarboxylation and the formation of a hemiacetal under argon. For each complex, two quantum yields were determined. Firstly, the quantum yield of the reduction from iron(III) to iron(II) was monitored spectroscopically using a phenanthroline derivative. The iron(II)phenanthroline complex exhibited a strong signal at 535 nm. Secondly, the quantum yield of the chelate cleavage was determined. This was achieved by monitoring the circular dichroism signal present in the intact chelate and its iron complexes. This method was used since chiral information was lost during the cleavage. The quantum yield associated with the reduction of iron(III) to iron(II) was determined to be Φ = 0.40, with the cleavage having the same value. A di‐μ‐oxo bridged iron(III) dimer was also prepared. Under the same conditions, the identical organic photochemical products were found. However, the quantum yield for the iron reduction was only half as high (Φ = 0.19), and the chelate cleavage was determined to be Φ = 0.12. It can be hypothesized that the lower quantum yield is primarily attributable to the oxo‐bridge's capacity to stabilize the Fe(III) oxidation state, since the dimer exhibits a lower reduction potential. Interestingly, the iron reduction quantum yield was about twice that of the chelate cleavage, suggesting that two Fe(III) centers are reduced per decarboxylation event. Next, a two‐electron oxidative decarboxylation occurred to obtain the hemiacetal. In the case of the oxo‐bridged iron(III) dimer, the electron is transferred from the remaining Fe(III) center. This shows that the di‐μ‐oxo bridged iron(III) dimer is less competent in the LMCT and has less productive follow‐up reactions than the homoleptic complex (Scheme 4A).^[^ ^38^ ^]^

The research group of McKenzie utilized a hexadentate ligand to investigate the behavior of various iron carboxylate complexes. Initially, they obtained a di‐μ‐oxo‐bridged complex as a solid‐state starting material. The complex was characterized by strong antiferromagnetic coupling between the high‐spin Fe(III) ions‐mediated by the oxo ligand. Upon over dehydration, the dimer could be cleaved into homoleptic complexes. The UV‐Vis spectroscopy studies showed that the homoleptic complexes depicted a local maximum at 360 nm. The dimer is cleaved into two diastereomers, the facial (*fac*‐) or meridional (*mer‐*) isomer. Interestingly, these mononuclear iron(III) complexes provide either a low‐spin (S = 1/2) or a high‐spin (S = 5/2) ground state.^[^ ^37^, ^39^ ^]^ This difference in spin states can often be attributed to the geometry and ligand field around the metal center. In both *fac*‐ and *mer*‐octahedral complexes, the arrangement of ligands can influence the splitting of the d‐orbitals. The *fac*‐isomer may have a ligand arrangement that leads to a stronger ligand field, resulting in a lower energy gap between the t2g and eg orbitals, thus favoring the low‐spin state (Scheme 4B).^[^ ^40^ ^]^ The high‐spin (S = 5/2) meridional diastereomer was identified to undergo photolytic CO_2_ release upon irradiation. Neither the low‐spin facial isomer nor the dimer appears to undergo LMCT.^[^ ^37^ ^]^

**Scheme 5:**
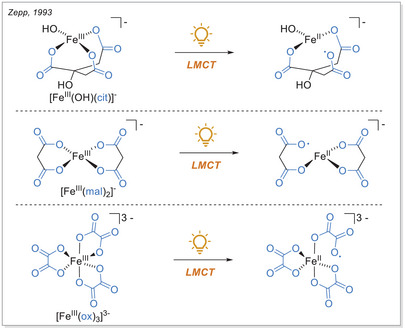
Photochemistry of aqueous iron(III)‐polycarboxylate complexes: exemplary complexes are shown. mal, malonate; cit, citrate.

Auxiliary ligands not only influence the quantum yield of the LMCT from iron to carboxylate, but the carboxylic acid itself also does so.^[^ ^28^, ^41^ ^]^ The Zepp research group investigated the photochemical redox reactions of iron(III) complexes with polycarboxylates (e.g., citrate, malonate, and oxalate) and their role in generating iron(II) in natural waters (Scheme 5). The group focused on determining the quantum yield for the formation of iron(II) from these complexes under various conditions, particularly pH and type of carboxylate. The Fe(III)‐citrate complex exhibited a decline in the quantum yield for Fe(II) formation, from 0.28 at pH 4 to 0.21 at pH 6 (436 nm), suggesting that higher pH conditions result in reduced quantum yields.

The quantum yield for Fe(III)‐malonate was found to be significantly lower at 0.027 (366 nm, pH 4), indicating that malonate is less effective than citrate in promoting the photochemical reduction of Fe(III) to Fe(II). Within the tested systems, the Fe(III)‐oxalate complex exhibited the highest quantum yield, with values of 1.0 for Fe(oxalate)^2−^ and 0.6 for Fe(oxalate)^3−^ (436 nm and pH 4). This demonstrates that oxalate is highly effective in facilitating the reduction of Fe(III) to Fe(II). This outcome demonstrates the efficacy of oxalate in facilitating the reduction of Fe(III) to Fe(II). The disparity in quantum yields among the various carboxylates can be ascribed to numerous factors, including the strength of the complexes formed with Fe(III), the stability of the resulting radicals, and the efficiency of the photochemical processes involved. For example, oxalate forms stronger complexes with Fe(III) and undergoes rapid photolysis, resulting in higher Fe(II) yields. In contrast, the weaker interactions and slower reaction kinetics associated with malonate result in lower quantum yields.^[^ ^35^ ^]^

### 2.3 Generation of Chlorine Radicals via LMCT of Fe(III)‐Cl Complexes

The tetrahedral [FeCl_4_]^−^ ion is known to exist in many organic solvents^[^ ^42^ ^]^ as well as in aqueous solution.^[^ ^43^ ^]^ The iron species obtained in aqueous solution are very different from those in aprotic organic solvents, these studies are outside the scope of this review.

**Scheme 6:**
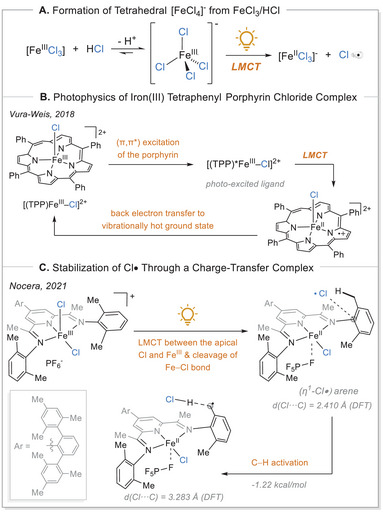
General overview of chloride radical formation after iron‐photoinduced LMCT. TPP, tetraphenylporphyrin.

A solution of anhydrous FeCl_3_ is known to undergo ligand redistribution reaction (FeCl_3_ → FeCl_4_^−^ + FeCl_2_^+^).The saturation with anionic chloride sources (LiCl, HCl, etc.) favors the formation of [FeCl_4_]^−^ ions (Scheme 6A).^[^ ^42^, ^44^ ^]^ The intensity of the characteristic absorption peaks associated with of [FeCl_4_]^−^ was monitored by time‐resolved UV‐Vis spectroscopy during irradiation. It was found that the bands at 360 nm and 310 nm gradually decrease in intensity upon prolonged near‐ultraviolet irradiation.^[^ ^42^, ^45^ ^]^ This suggested that [FeCl_4_]^−^ is being converted into iron(II) and the chlorine radical, and therefore being photochemically active species (Scheme 6). The DFT analysis revealed that the charge transfer occurs primarily from the chloride anionic ligands to the iron center (24.8% each, 99.2% in total), indicating that the excitation of [FeCl_4_]^−^ occurs via the LMCT process.^[^ ^42^, ^44^, ^46^ ^]^

Interestingly, the choice of solvent is crucial for the photochemical performance of the complex, since the autoionization needs to be rationalized as a more complex mixture (FeCl_3_ + solvent→ FeCl_4_^−^ + Fe(solvent)_0–4_Cl_2_^+^).^[^ ^42^, ^46^, ^47^ ^]^ The UV‐spectra in weakly coordinating solvents such as acetonitrile, toluene or chloroform depict vastly different maxima than those in strongly coordinating solvents like alcohols or pyridine.^[^ ^42^, ^46^ ^]^ Using strongly coordinating solvents like methanol diminishes the generation of chloride radicals as the lifetime of the dominant transient species identified by transient absorption spectroscopy is reduced to about 100 fs. Weaker coordinating solvents like acetonitrile, toluene or chloroform show two absorption bands, in which three‐fourths of the excited state of iron undergoes charge recombination to the ground state, but the remaining one‐fourth of the excited state converts into a long‐lived species (>2 ns).^[^ ^46^ ^]^ This long‐lived species can be assigned to the iron to chloride charge transfer.^[^ ^12^, ^27^, ^46^, ^48^ ^]^

A variety of different auxiliary ligands were investigated, and we would like to emphasize the importance of the iron complexes undergoing LMCT to form chloride radicals and discuss the ligand's influence on this process.^[^ ^49^ ^]^ A number of studies have been conducted which investigated the photochemical processes in iron porphyrin complexes. The time evolution of iron(III) tetraphenyl porphyrin chloride under photoirradiation was monitored by means of transient absorption spectroscopy. The research conducted by Vura‐Weis group has revealed that, in the initial phase, the π → π* transition of the complex's ligand was excited by light exposure.^[^ ^49^ ^]^ This excited state then evolves to a LMCT state, thereby changing the oxidation state from iron(III) to iron(II). Despite the binding of chloride to the complex, the LMCT occurs between the porphyrin and the iron, thereby preventing the generation of a chloride radical. Two decay mechanisms have been postulated for the ultrafast relaxation of iron(III) porphyrins. The first involves direct relaxation to a vibrationally hot ground state, in which one of the paired electrons from the metal d orbital is transferred to the porphyrin π‐system, generating a high‐spin Fe(III) species (S = 5/2). The second pathway proceeds via a metal‐centered excited‐state intermediate, where an unpaired electron from a low‐lying d‐orbital is transferred to the porphyrin π‐system, producing a low‐spin (d,d) Fe(III) excited state (S = 3/2) that subsequently relaxes to the ground state. Vura‐Weis and co‐workers demonstrated that the LMCT state relaxes to a vibrationally hot ground state within 1.13 ps, without involvement of (d,d) intermediates. The use of a tabletop extreme‐ultraviolet probe, together with semiempirical ligand field multiplet (LFM) calculations, enabled unambiguous assignment of metal‐ versus ligand‐centered excited states and resolved longstanding discrepancies in the relaxation pathways of Fe(III) porphyrins (Scheme 6B).^[^ ^49^ ^]^

The Nocera research group introduced a pyridine(diamine) (PDI) ligand (Scheme 6C). In this system, the LMCT from chloride to iron(III) occurs, followed by the homolytic cleavage of the Fe(III)─Cl bond and the generation of chlorine radical. This particular supporting ligand enables the stabilization of the reactive chlorine radical within a secondary coordination sphere. The PDI‐based ligands induce regioselectivity through confinement of the chlorine radicals within the secondary coordination sphere via the formation of a Cl•(arene) complex with the arene moieties present in the PDI‐based ligands. This allows the selectivity problem to be tackled, as selectivity control is no longer dictated only by the substrate stereoelectronics.^[^ ^50^ ^]^

## 3 Mechanistic Insights Into Light‐Induced Iron‐Catalyzed Transformations via LMCT

In the field of photochemistry, the iron‐photoinduced LMCT approach has extensively applied for organic transformations; however, the in‐depth mechanism of a reaction has rarely been studied. The synthetic community primarily focuses on developing new reactivities, and unfortunately, little emphasis is placed on understanding the underlying mechanisms. That may be due to unfamiliarity with analytical methods required for open‐shell transformations. The investigation of reaction mechanisms is particularly challenging, as it necessitates the consideration of classical conditions such as temperature and concentration, in addition to numerous additional factors associated with light, including wavelength, distance, light intensity, power, and so forth. In this article, however, we would like to convey that the effort to investigate the mechanism can be worthwhile and new findings open the avenue of reactivities under iron‐photoinduced LMCT. Furthermore, investigating the mechanism also enables a more rational approach to establishing optimal reaction conditions. A fantastic starting point for mechanistic studies can be found in reviews by Melchiorre^[^ ^51^ ^]^ and Llyod‐Jones.^[^ ^52^ ^]^ The synthetic utility of this field has increased dramatically in recent years, yet mechanistic insights are still scarce. Five recently published works with in‐depth mechanistic studies are highlighted here.

### 3.1 Decarboxylative Giese‐Type Reaction

The Ackermann‐Biegasiewicz group has conducted a detailed study on direct decarboxylative Giese‐type additions using LMCT‐mediated iron‐photocatalyzed approach. A key focus of their investigation was the effect of various commercially available ligands on the reaction outcome. They observed that aliphatic amine ligands, particularly diethylenetriamine, significantly enhanced the reaction efficiency, which is particularly notable given their typical susceptibility to photodegradation via direct oxidation or C─H bond abstraction at α‐nitrogen positions.^[^ ^53^ ^]^ Interestingly, this approach demonstrated superior efficiency and broader substrate scope compared to previous work by Jin and co‐workers,^[^ ^54^ ^]^ where pyridine‐type ligands were primarily utilized, often leading to lower reactivity and limited tolerance to functionalized carboxylic acids. The unusual observation that ligand choice had such a profound influence on the transformation prompted the authors to investigate the mechanism in depth through UV‐Visible spectroscopy, thermogravimetric analysis (TGA), and density functional theory (DFT) calculations.They revealed how these aliphatic amine ligands facilitate more efficient LMCT activation while stabilizing reactive intermediates, thereby suppressing undesired side reactions. This study highlights how strategic ligand design can dramatically improve iron‐photocatalyzed decarboxylation processes, offering a robust platform for alkyl radical generation under mild and sustainable conditions.

**Scheme 7:**
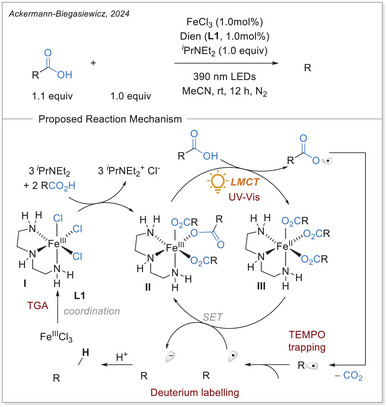
Direct decarboxylating Giese‐type reaction and the proposed mechanism. Dien, diethylenetriamine.

The proposed mechanism of the Giese‐type reaction with the ligated iron was evaluated (Scheme 7). A formation of the precatalyst **I** was postulated, which is over ligand exchanged transferred into the active catalyst **II**. After excitation and homolytic cleavage of the iron carboxylate bond the reduced complex **III** is formed. The formed so‐generated carboxyloxyl radical lose the carbon dioxide and the alkyl radical is obtained, which subsequently adds to the Michael acceptor. The formed carbon‐centered radical is reduced by the iron(II) complex **III**, which with another coordination of a carboxylate leads back to the active catalyst **II**. Finally, the hydroalkylation product is obtained through protonation.^[^ ^53^ ^]^ This prosed mechanism is in agreement with previous publication by the Jin working group under similar reaction conditions.^[^ ^54^ ^]^

The formation of precatalyst **I** was determined with the help of TGA. Here, the in‐situ generated complex is compared to the individual components. A delay in thermal degradation and a weight percentage loss that can be attributed to ligand loss. With comprehensive UV‐Vis studies, the species **II** was identified as photochemically active by comparing the absorbance of the in‐situ generated complex with that of precatalyst **I** and with the absorbance properties of Fe(OAc)_2_ and ligand. To prove photochemically induced decarboxylation, instead of thermal, light on‐off experiments were performed to reveal no proceeding of reaction without light irradiation. Through TEMPO trapping experiments, a radical pathway was uncovered. Finally, it was postulated whether the resulted carbon‐centered radical after addition to the Michael acceptor is either quenched through HAT from C─H bond (solvent or base) or being reduced by iron(II) as a reducing agent and subsequent protonation. To examine this, deuterium labelling experiments were carried out with deuterated base or deuterated solvent, no deuterium incorporated product was observed. When deuterium source was added in the form of a proton equivalent (RCOOD and/or D_2_O), incorporation of deuterium in the product was observed. It is evident that the pathway via reduction and subsequent protonation is significantly more pronounced than the HAT pathway.^[^ ^53^ ^]^

### 3.2 Iron‐Catalyzed Decarboxylative Oxygenation With TEMPO

**Scheme 8:**
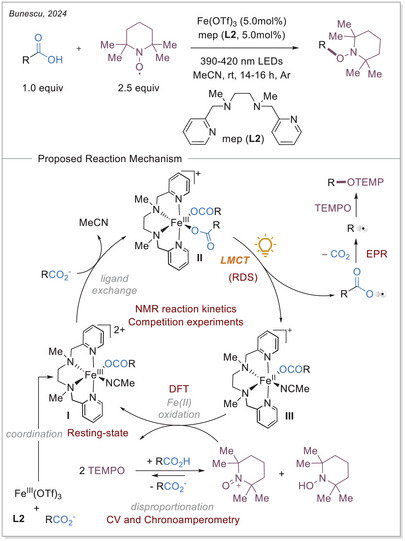
Decarboxylating oxygenation of aliphatic carboxylic acids and the proposed reaction mechanism.

The research groups of Bunescu^[^ ^55^ ^]^ and Guérinot^[^ ^56^ ^]^ simultaneously published a decarboxylative oxygenation using 2,2,6,6‐tetramethylpiperidin‐1‐yl)oxyl (TEMPO), demonstrating broad tolerance toward carboxylic acids and diverse functional groups. Given the similarity of both reports, we discuss in this section the contribution from Bunescu and co‐workers, which offers a more detailed mechanistic perspective. As mechanistic studies on Fe‐photocatalyzed transformations remain scarce in this emerging area, their work represents an important advance. Through a combination of reaction kinetics, electrochemistry, EPR, UV‐Vis, HRMS, and DFT calculations, the authors provided a comprehensive validation of the proposed mechanism (Scheme 8).

The catalytic cycle for the Fe‐catalyzed decarboxylative oxygenation consists of in‐situ formation of the resting state **I**, through ligation of **L2** and carboxylate. The coordination of an additional carboxylate facilitates the formation of the active photochemically‐active complex **II**. This complex undergoes photoexcitation and subsequent LMCT/homolysis sequence resulting in the formation of a carboxyloxyl radical. The carbon dioxide loss furnishes the carbon‐centered radical which is then trapped by TEMPO. The oxidation of iron(II) to iron(III) completes the catalytic cycle. The iron(II) complex **III** is oxidized by TEMPO^+^, which is generated in‐situ via acid‐catalyzed disproportionation of TEMPO to TEMPO^+^ and TEMPOH.

Primarily, kinetic studies were carried out for each reaction component, showing that carboxylic acid, catalyst (**L2**•Fe(OTf)_3_), and light intensity are of the first order. However, TEMPO was determined with zero reaction order, suggesting that the rate‐determining step (RDS) takes place before the reoxidation of iron(II). A combination of competition experiments and quantum yield measurements identified the photoinduced carbon‐centered radical generation via LMCT as the rate‐limiting step (RDS).

Next, the reoxidation of the iron(II) to iron(III) species was extensively studied. The redox potential of Fe^III^/Fe^II^ couple for the in‐situ generated **L2•**Fe(OTf)_2_ complex was found to be very high (1.1 V versus Ag/AgCl in acetonitrile) but decreased significantly when Fe(OAc)_2_ is used instead of Fe(OTf)_2_ (0.16 V versus Ag/AgCl in acetonitrile). Conversely, over measurements of TEMPO, for which E_1/2 _= 0.74 V (versus Ag/AgCl in acetonitrile), it was deduced that the oxidation of **L2**•Fe^II^(OCOR)_2_ by TEMPO via electron transfer (ET) or proton‐coupled electron transfer (PCET) is thermodynamically unfavorable. Chronoamperometry was utilized to established the disproportionation of TEMPO under acidic conditions, suggesting that TEMPO⁺ serves as the actual oxidant rather than TEMPO. The formation of iron(II) upon irradiation of the in‐situ generated iron(III) complex **II** was also confirmed. Additionally, EPR spectroscopy once again confirmed that TEMPO⁺ acts as the oxidizing species for iron(II). Furthermore, by illuminating and simultaneously freezing the sample, the carbon‐centered alkyl radical generated after the decarboxylation could be detected.

### 3.3 Decarboxylative Alkylation via Imidazole‐Coordinated Iron‐Clusters

**Scheme 9:**
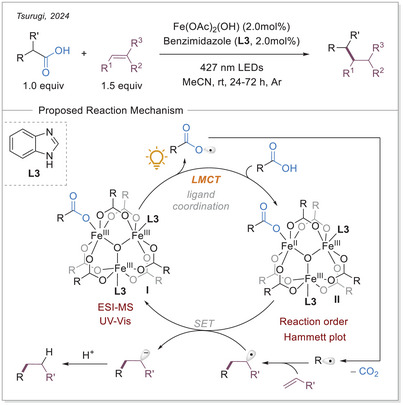
Decarboxylative alkylation of alkenes and the proposed reaction mechanism.

In 2024, Tsurugi research group utilized imidazole‐coordinated iron clusters to undergo decarboxylative alkylation of various alkenes. The study emphasizes the use of iron(III) complexes, particularly with the ligand benzimidazole (**L3**), which significantly enhances the catalytic performance. The coordination of **L3** has been shown to increase the absorption coefficient in the visible light region, thereby enhancing the photo‐responsivity for the homolytic cleavage of the iron(III)‐carboxylate bond (Scheme 9).^[^ ^57^ ^]^

In the proposed mechanism, a trinuclear iron(III)‐cluster I is formed in‐situ from iron(III) acetate, carboxylic acid, and benzimidazole (**L3**). Upon irradiation, it undergoes cluster homolysis, resulting in the formation of an iron(II)‐containing cluster (B) and a carboxyloxyl radical. After subsequent decarboxylation and addition to the Michael acceptor, the generated radical oxidizes the iron(II) center of the cluster **II** to obtain the iron(III) cluster **I**.

The iron(III)‐clusters were characterized by means of electrospray ionization mass spectrometry (ESI‐MS) and UV‐Vis spectroscopy. Basic iron(III) acetate was mixed with excess amounts of acid and ligand **L3**, resulting in the observation of the formation of predominantly trinuclear iron(III) clusters. In the absence of the external ligand **L3**, a multitude of iron clusters was observed. The reaction performed significantly better with the addition of **L3**, and the trinuclear iron(III) cluster was identified as photochemically active. In kinetic studies, first order was established in Michael acceptor and light intensity. However, zeroth order was found in the catalyst and the carboxylic acid. This finding emphasizes that the addition of radical into the Michael acceptor is the RDS. Furthermore, the lack of rate dependence on the acid concentration and catalyst indicates rapid photochemical generation of the carboxyloxyl radical. A competition experiment was conducted using substituted aryl acetic acids to perform a Hammett plot, which indicated that electron‐rich substrates reacted faster than electron‐deficient ones. This finding is consistent with the proposed reaction mechanism, as it is expected that the more nucleophilic radical intermediates will add faster to the electron‐poor Michael acceptor.

### 3.4 Aerobic Carbonylation of Methane

**Scheme 10:**
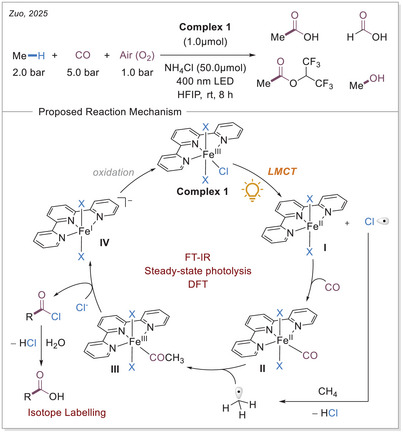
Fe‐catalyzed Aerobic carbonylation of methane via LMCT and the proposed reaction mechanism.

In 2025, an iron‐catalyzed aerobic carbonylation of methane was published by the research group of Zuo. This approach allows for exceptional selectivity in carbonylation reactions under mild conditions, overcoming the challenges associated with the sluggish reactivity of methyl radicals and carbon monoxide (Scheme 10).^[^ ^58^ ^]^

In the proposed mechanism, the iron(III) complex (**Complex 1**) is photochemically activated, and under homolysis, the iron(II) complex II and the chlorine radical are generated. Subsequently, HAT by the chlorine radical from methane forms the methyl radical. The association of carbon monoxide to iron(II) complex **I** results in the formation of iron(II) carbonyl complex **II**. The outer‐sphere addition of methyl radicals to the carbonyl ligand of complex **II** provides the acetyl iron(III) complex **III**. Following the formation of C(O)─Me bond, the acetyl iron(III) complex reacts with chloride anions to form acetyl chloride and low‐valent iron(I), which is then hydrolyzed to furnish acetic acid.

Fourier‐transform infrared spectroscopy (FTIR) was used to investigate the mechanism. The formation of the iron(II) carbonyl complex **II** (at 1968 cm^−1^) was monitored in comparison to the CO stretching vibration (at 1650 cm^−1^). Steady‐state photolysis of the iron complexes was conducted to observe the formation of reduced iron(II) species and to validate the LMCT‐mediated HAT reactivity. The formation of the methyl radical was confirmed via trapping experiments with an electron deficient olefin. A considerable endeavour was undertaken to acquire a comprehensive series of isotope‐labeled experiments. The initial carbon labelling of the carbon monoxide (^13^CO) revealed that the carbon monoxide is incorporated into the carboxylate. The labelling of methane (^13^CH_4_) provided the necessary information to ascertain that it is incorporated as the acetate group. In addition, the oxygen labelling demonstrated that the formation of the carboxylic acid occurred in the presence of heavy water (^18^OH_2_). A reaction energy profile was also established via DFT studies. It was shown that the carbon monoxide coordination to iron takes place first, followed by the addition of the methyl radical to iron‐bound carbon monoxide.

### 3.5 Photoinduced Chlorine Radical‐Mediated C─H Activation

**Scheme 11:**
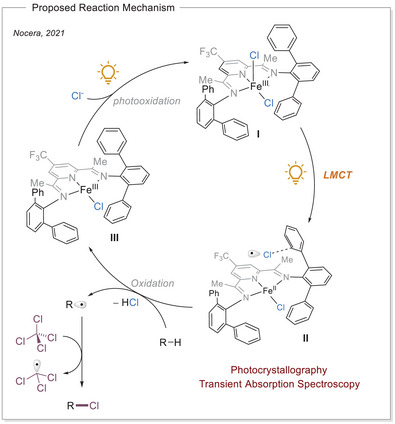
Proposed reaction mechanism for photocatalytic C − H functionalization by iron(III) chloride − pyridine(diamine) complex.

The research group of Nocera published a C─H functionalization reaction of aliphatic C─H bonds via HAT with chlorine radicals generated via LMCT from iron(III)‐chloride complex. This system allows the selective chlorination or bromination of the starting material depending on what type of halogenation reagent is employed (Scheme 11).^[^ ^59^ ^]^

The free chlorine radical is formed via LMCT of iron(III) chloride complex during photoirradiation. However, the chlorine radicals are highly reactive and usually result in low regioselectivity when multiple aliphatic C─H bonds are present. To address this, they introduced a PDI‐based ligand that allows the stabilization of the reactive chlorine radical within a secondary coordination sphere. The PDI‐based ligands induce regioselectivity through confinement of the chlorine radicals within the secondary coordination sphere via the formation of a Cl•arene complex with the arene moieties present in the PDI‐based ligands. This allows the selectivity problem to be tackled, as selectivity control is no longer limited to the substrate.^[^ ^50^, ^59^ ^]^ The complex forms intramolecular Cl•arene CT complexes. Due to electronic stabilization of Cl• by the aromatic ring (∼3.0 kcal/mol for Cl•benzene with respect to Cl• in solution), the Cl•arene CT complexes were observed over transient absorption, and determined by single‐crystal X‐ray diffraction.^[^ ^50^ ^]^

The proposed mechanism involves the irradiation of iron(III) complex **I**, which results in the formation of chlorine radical and iron(II) complex **II** via LMCT. Subsequently, the chlorine radical engages in a charge transfer complex with the aromatic ring of the ligand, located within the secondary coordination sphere of the complex. Thereafter, the stabilized radical abstracts a hydrogen atom from a proximate substrate, leading to the formation of hydrochloric acid. The carbon‐centered radical is then saturated via XAT from the radical trap. The photochemical oxidation of the iron(II) PDI complex by nitromethane, in conjunction with the addition of a chloride ligand, results in the regeneration of the initial iron(III) complex, thereby closing the catalytic cycle.^[^ ^59^ ^]^

The mechanism was investigated initially with femtosecond‐resolved TA, during which two new species were identified after pumping with 360 nm light. One of the new species is attributed to the Cl•arene complex and the other to the iron(II) complex **II**. Furthermore, photo crystallographic experiments were conducted. A single crystal of the complex **I** obtained from DCE was irradiated for 83 minutes with 450 nm light at 15 K. The experimental setup allows for the observation of structural changes in the crystal as a result of light exposure. The measurements demonstrated photochemical activation of the apical Fe─Cl bond, with photoinduced structures exhibiting a gradual increase in occupancy as the crystal was irradiated. This finding indicates that the reaction between the photo‐eliminated chlorine radicals and the DCE solvent leads to the accumulation of photoproducts over time. Overall, the studies highlight a secondary coordination sphere strategy that enhances the selectivity of chlorine radicals in C─H functionalization reactions, expanding the methodologies available in organic photocatalysis.

## 4 Photoactivation of Various Bonds Through Iron‐Photoinduced LMCT: Synthetic Applications

The topic of LMCT‐driven iron photocatalysis has gained significant attention in recent years and has become a active area of research in chemistry, as highlighted in several comprehensive reviews.^[^ ^60^ ^]^ This section provides a systematic overview of the synthetic applications of iron‐photoinduced LMCT strategy, categorized by the type of iron–L (L = halide, carboxylate, alkoxy, and azide) species exhibiting LMCT reactivity.

### 4.1 LMCT of Halogen‐Iron Species

#### 4.1.1 Substrate Activation via HAT by Halo Radicals

Both Fe(III)─Cl and Fe(III)─Br complexes are capable of generating radicals. Among them, chlorine radicals (Cl•) exhibit higher reactivity, while bromine radicals (Br•) offer better selectivity. Due to their strong oxidizing nature and ability to efficiently generate radicals, Fe(III)─Cl species serve as key intermediates in the LMCT process. This process liberated chlorine radicals, which subsequently participated in HAT with the target C─H bonds, leading to the formation of carbon‐centered radicals. These reactive intermediates can further participate in bond‐forming reactions, facilitating the construction of C─C, C─N, C─O, and C─X bonds, among others.^[^ ^61^ ^]^ Such transformations eliminate the need for pre‐functionalization and enable the efficient post‐functionalization of organic molecules, contributing to the synthesis of valuable compounds in pharmaceuticals, fine chemicals, and other industries.

Given their crucial role in radical generation and subsequent transformations, this chapter will primarily focus on chlorine radicals in iron‐catalyzed photochemical reactions via LMCT process.

##### C─C bond formation via HAT

C─C bond formation is at the foundation of organic synthesis as it enables the construction of complex molecular frameworks found in natural products, pharmaceuticals, and functional materials. In particular, the addition of carbon‐centered radicals into electron‐deficient alkenes to form C─C bonds is referred to as the Giese‐type reaction.^[^ ^62^ ^]^ The obtained α‐carbon radical can further engage in a tandem process with a second electrophile, expanding the reaction's synthetic utility.

In 2021, two independent studies conducted by Duan^[^ ^63^ ^]^ and Rovis^[^ ^64^ ^]^ independently reported photocatalytic processes involving C─H alkylation and amination reactions of unactivated alkanes. Remarkably, all investigations employed LMCT to induce homolysis of the Fe─Cl bond in FeCl_3_ under similar reaction conditions.

**Scheme 12:**
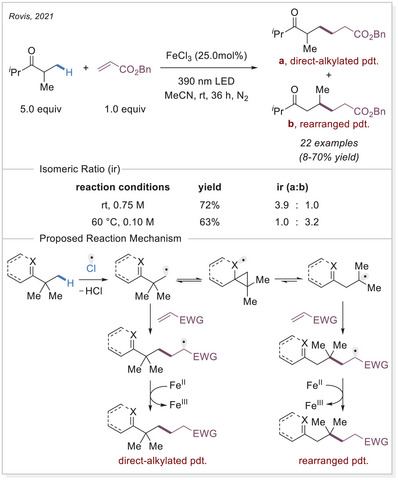
Iron‐catalyzed skeletal rearrangement and C─H alkylation via photoinduced LMCT.

Rovis group reported a strategy for promoting alkyl group migration and C─H alkylation (Scheme 12).^[^ ^64^ ^]^ Typically, C─C bond cleavage requires high‐energy intermediates, making it challenging. However, in their approach, the generation of unstable alkyl radicals, which are formed through the abstraction of an electron‐rich C(sp^3^)─H bond by a chlorine radical, enables 1,2‐alkyl migration in the presence of suitable functional groups. The resulting radical undergoes addition to an electron‐deficient olefin, recombines with an iron(II) complex, and undergoes protodemetalation, leading to alkylation and regeneration of iron(III) catalyst. Simple modifications of the reaction conditions allow for the selective synthesis of the directly alkylated or the rearranged‐alkylated products. Increasing the temperature to 60 °C and decreasing the concentration to 0.10 M favors the rearrangement pathway.

**Scheme 13:**
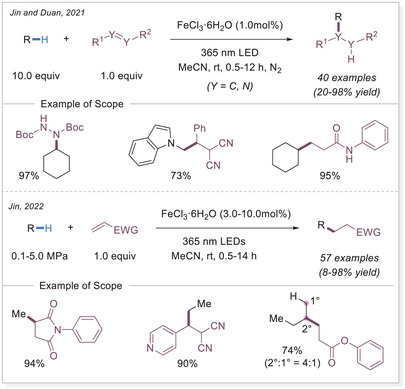
Iron‐catalyzed C(sp^3^)─H functionalization to form C─N and C─C bonds.

On the other hand, Duan and Jin published two independent works using FeCl_3_•6H_2_O to convert C─H bonds into C─C or C─N bonds under light irradiation (Scheme 13). Initially, they showcased the successful substitution of C(sp^3^)─H bond with di‐*tert*‐butyl azodicarboxylate derivatives under 365 nm light irradiation at room temperature.^[^ ^63^, ^65^ ^]^ According to their proposed mechanism, LMCT generates active chlorine radicals, which facilitate a HAT process to form alkyl radicals. These radicals are subsequently trapped by an unsaturated bond, leading to the formation of a nitrogen‐centered radical. Finally, the nitrogen radical oxidizes the iron species and undergoes protonation to yield the desired product. In a subsequent study,^[^ ^65^ ^]^ Jin further expanded this approach by using methane as a substrate. This reaction is of particular significance due to methane's strong C─H bonds, which make it notoriously difficult to activate. The functionalization scope of ethane and other light alkanes was also explored and worked with good yields.

**Scheme 14:**
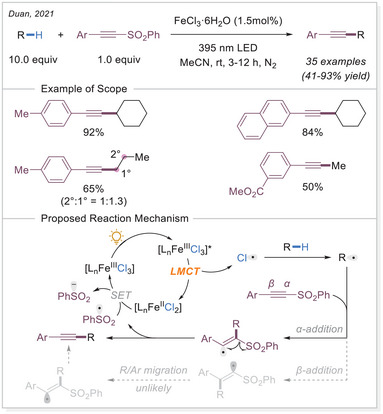
Photo‐induced direct C(sp^3^)─H alkynylation by iron catalysis.

Aside from double bond modification, Jin, Duan, and coworkers expanded their approach to C(sp^3^)─H alkynylation using iron chloride (Scheme 14).^[^ ^63^ ^]^ Their method proved effective, enabling the coupling of alkynes with alkyl radicals, followed by a radical‐fragmentation to remove the sulfone group. Notably, the protocol demonstrated broad applicability, accommodating various liquid alkanes as well as more challenging gaseous alkanes, including methane and ethane.

**Scheme 15:**
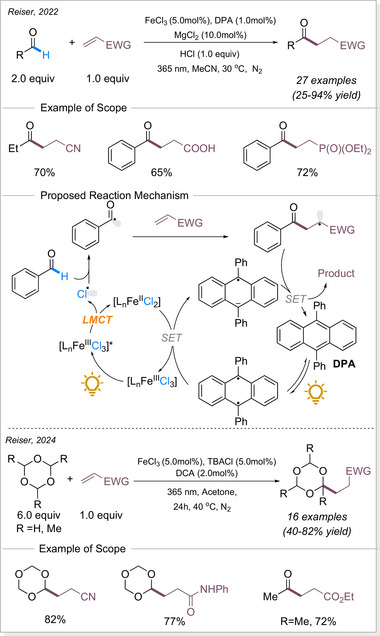
Iron‐catalyzed light‐induced C─H bond functionalization of aldehydes. DPA = 9,10‐diphenylanthracene. DCA = 9,10‐dicyanoanthracene.

Not only C(sp^3^)─H bonds but also aldehyde C─H bonds can undergo functionalization. In 2022, Reiser's group reported a similar Giese‐type reaction using aldehydes as substrate (Scheme 15).^[^ ^66^ ^]^ They developed a dual catalytic strategy by utilizing FeCl_3_ as a chlorine radical source in combination with the redox mediator 9,10‐diphenylanthracene (DPA) as a co‐photocatalyst, allowing the hydroacylation of electron‐deficient alkenes. DPA acts as a redox mediator to facilitate the regeneration of Fe(III), while MgCl_2_ serves as a Lewis acid activation of the acceptor alkene. The Reiser group later developed a dual‐photocatalytic protocol using iron(III) chloride and 9,10‐dicyanoanthracene (DCA) for the alkylation of Michael acceptors via HAT with 1,3,5‐trioxanes. This approach enables versatile access to masked hydroformylation, hydroacylation, and hydrocarboxylation products.^[^ ^67^ ^]^

**Scheme 16:**
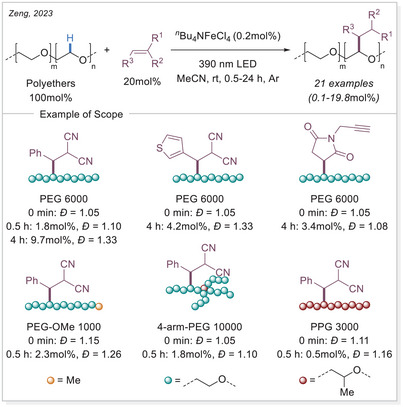
C─H functionalization of polymers via iron photocatalysis.

Iron photocatalysis via LMCT has also been applied to the C─H functionalization of polymers. Polyethylene glycols (PEGs) are widely used in surface modification, biofunctionalization, drug delivery, hydrogels, and solid‐state electrolytes. However, achieving the selective C─H functionalization of PEGs remains challenging due to significant obstacles such as degradation and gelation. In 2023, Zeng's group developed a catalytic strategy for modifying PEGs via C─H radical alkylation with electron‐deficient alkenes using tetrabutylammonium iron(III) chloride (*^n^* Bu_4_NFeCl_4_) as catalyst under irradiation, successfully preserving molecular weight (MW) and polydispersity index (Scheme 16).^[^ ^68^ ^]^

The level of functionalization (LOF) could be controlled by varying the number of alkenes and reaction time, while maintaining a narrow molecular weight distribution. A proportional growth in the LOF was observed with an increase in the starting alkenes, until it reached the maximum LOF in 19.8mol% after 50 h. In the case of polymer post‐functionalization strategies, it is crucial to sharpen the MW distribution (MWD) and diminish the degradation for achieving uniform polymer properties and enhanced performance control. The narrow dispersity (*Đ*) values could be achieved by simply lowering the reaction concentration to 0.5 or 0.2 M. A broad scope of electron‐deficient alkenes containing nitrile, ester, epoxide, terminal alkynyl, 2,5‐dioxotetrafuranyl, and 2,5‐dioxopyrrolidinyl groups could be utilized to functionalize the different polyethers with great efficiencies. This method provides an effective approach for incorporating diverse functional groups along the PEG backbone.

**Scheme 17:**
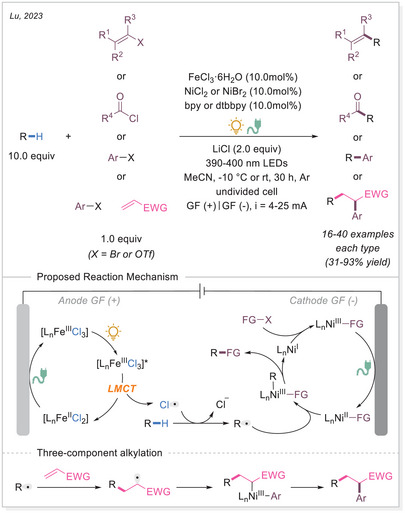
Photoelectrochemical C(sp^3^)─H alkenylation/arylation/alkylation using a binary catalytic system. bpy, 2,2′‐bipyridine; dtbbpy, 4,4′‐di‐*tert*‐butyl‐2,2′‐bipyridine; GF, graphite felt.

In 2023, Lu's group integrated electrochemistry into photoinduced LMCT processes by employing dual catalysis at both electrodes, enabling the alkenylation and acylation of alkanes (Scheme 17).^[^ ^69^ ^]^ Later that year, they applied the same strategy to achieve C─H arylation and selective alkylation.^[^ ^70^ ^]^ Aryl bromides served as the C(sp^2^) coupling partners, reacting with nickel species to form aryl─Ni(II) intermediates generated at the cathode. Notably, when an alkene was introduced as a linker, a three‐component C(sp^3^)─H alkylation could be achieved. Notably, current density played a crucial role in determining product selectivity by accelerating the formation of aryl─Ni(II) species. At a higher current (25 mA), the carbon‐centered radical preferentially underwent irreversible Giese‐type addition, outcompeting the alkyl radical metalation pathway. This work demonstrates a tunable selectivity switch between two‐component C(sp^3^)─H arylation and three‐component C(sp^3^)─H alkylation of alkanes via paired oxidative and reductive catalysis.

**Scheme 18:**
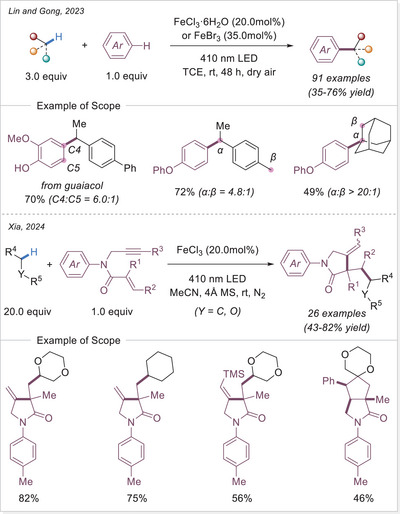
Iron‐catalyzed photo‐induced C(sp^2^)─H/C(sp^3^)─H coupling of arenes with hydrocarbons and radical cascade via C(sp^3^)─H alkylation for the synthesis of γ‐lactams. TCE, 1,1,2,2‐tetrachloroethane.

In 2023, Lin and Gong's group developed a direct and selective coupling of electron‐rich arenes with aliphatic hydrocarbons, yielding a wide range of products with excellent chemo‐ and site‐selectivity (Scheme 18, top).^[^ ^71^ ^]^ It is proposed the generated carbon‐centered radicals would transform to carbocations via SET and then undergo Friedel‐Crafts‐type process. Iron halides served as efficient catalysts, promoting high regioselectivity even at challenging C(sp^2^)─H positions. For example, benzylation of guaiacol occurred preferentially at the C4‐ and C5‐positions with a 6.0:1 ratio. In the case of C(sp^3^)─H activation, arylation favored secondary C─H bonds over primary or tertiary ones, probably guided by a balance of sterics, electronics, and iron coordination effects. Notably, adamantane and its derivatives possess stronger tertiary C─H bonds compared to their secondary counterparts, the reaction exhibited exclusive selectivity for tertiary C─H bonds, highlighting the unique reactivity profile of this iron‐catalyzed system.

The synthesis of biologically active compounds can also benefit from this approach. Cascade radical cyclization of 1,n‐enynes has emerged as a powerful strategy for constructing both carbon and heterocyclic ring systems. Among these, γ‐lactams have drawn considerable attention due to their potential activity against penicillin‐binding proteins (PBPs). When a cascade process is applied to the γ‐lactam ring, a five‐membered ring can readily form via a 5‐intro cyclization from 1,6‐enynes or 1,6‐olefins. In 2024, Xia's group reported a C(sp^3^)─H alkylation reaction via photo‐induced LMCT process, forming alkylated γ‐lactam ring from 1,6‐enyne with unactivated alkanes and ethers during cascade cyclization process (Scheme 18, bottom).^[^ ^72^ ^]^ In this reaction system, alkyl radicals were generated and added to the olefin terminus of 1,6‐enyne, triggering the ring‐closing event, producing alkylated γ‐lactam derivatives with yields ranging from 43–92%. Interestingly, when terminal substituent R^3^ was a hydrogen atom or a trimethylsilyl group, a single pentagonal ring product with tandem addition was obtained. However, when R^3^ was a methyl or phenyl group, products with a spiral ring through further [2 + 2 + 1] cyclization process were also obtained, in moderate yields. The ratio of spiral ring products and single five‐membered ring products is about 4.5:1.

**Scheme 19:**
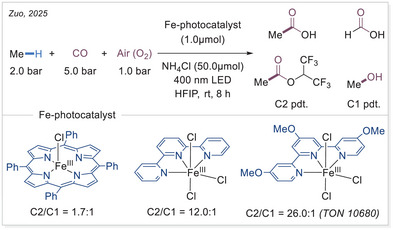
Photocatalytic aerobic carbonylation of methane. HFIP, hexafluoro‐2‐propanol.

Despite the inertness of the C─H bond in methane, its direct transformation into valuable chemicals remains a highly pursued goal in modern chemistry. In 2025, Zuo's group reported a breakthrough using an iron‐terpyridine catalyst system that leverages LMCT to achieve high C2/C1 selectivity of carbonylation (Scheme 19).^[^ ^58^ ^]^ They found that increasing the catalyst loading at constant CO pressure enhanced C2/C1 selectivity, as did raising the CO pressure at constant catalyst loading. Interestingly, iron catalysts bearing electron‐withdrawing groups (e.g., carboxylic ester or trifluoromethyl) led to reduced efficiency and selectivity for acetic acid formation. In contrast, catalysts modified with electron‐donating groups such as *tert*‐butyl or methoxy displayed improved selectivity, highlighting the influence of ligand electronics on catalytic performance. For the detailed mechanism of this transformation, see Section 3.4 of this review.

**Scheme 20:**
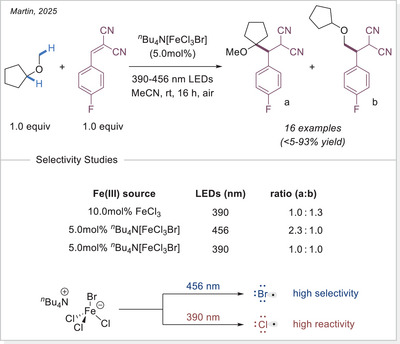
Wavelength‐dependent selectivity studies with different iron sources.

In 2025, Martin's group reported a LMCT‐driven C─H functionalization strategy using mixed iron halide catalysts and investigated their reactivity under different wavelengths of light (Scheme 20).^[^ ^73^ ^]^ While the reactions initiated under 456 nm blue light proceeded more slowly compared to those under 390 nm irradiation, they displayed notable differences in site‐selectivity and radical behavior. Mechanistic studies revealed that excitation of the [FeCl_3_Br]^−^ anion at 456 nm primarily generated bromine radicals via LMCT, which are known for their higher selectivity in HAT processes. In contrast, 390 nm irradiation predominantly produced chlorine radicals, which are significantly more reactive but less selective. This study provides a valuable framework for tuning radical reactivity in iron‐catalyzed photochemical transformations through wavelength control.

##### C─N bond formation via HAT

**Scheme 21:**
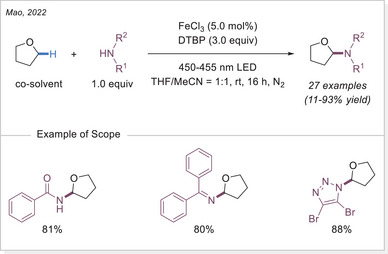
Iron‐catalyzed N─H alkylation of amides and *N*‐heterocycles. DTBP, di‐*tert*‐butyl peroxide.

The efficient formation of C─N bonds plays a crucial role in organic synthesis, as these bonds are fundamental structural units in natural products, pharmaceuticals, and advanced materials. In 2022, Mao and coworkers reported a Fe(III)‐catalyzed N─H alkylation of amides and *N*‐heterocycles under visible light using an iron photocatalyst in combination with the HAT process, with an external oxidant, di‐tert‐butyl peroxide, to facilitate the reaction (Scheme 21).^[^ ^74^ ^]^ However, substrates other than THF, including light alkanes, ethers, and toluene, all failed to provide the corresponding products.

**Scheme 22:**
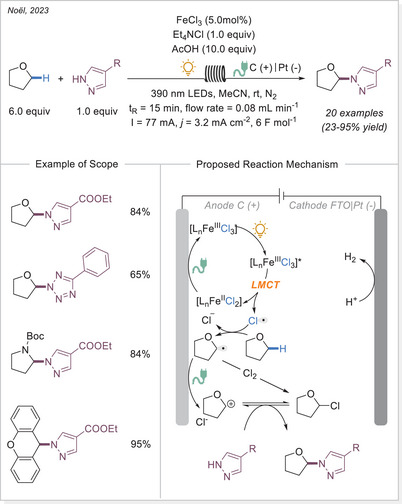
C(sp^3^)─H heteroarylation conducted in a flow photoreactor, undergoing LMCT process combined with electrocatalysis. FTO, fluorine‐doped tin oxide.

Expanding on previous studies on HAT photocatalysis using decatungstate,^[^ ^75^ ^]^ Noël^[^ ^76^ ^]^ and colleagues^[^ ^74^ ^]^ introduced the photoelectrochemical process in a continuous flow set‐up in 2023 (Scheme 22). Their novel flow reactor simultaneously accommodates both photons and electrons in the microchannel, enabling the control of transient species. This oxidant‐free strategy integrates photoinduced LMCT with electrochemical anodic oxidation, ensuring efficient iron catalyst regeneration while promoting the oxidation of α‐oxy radical intermediates. This method exhibits a wide substrate scope, enabling the selective incorporation of various nitrogen‐containing heterocycles at the α‐position of ethers, offering a sustainable approach to C(sp^3^)─H heteroarylation.

**Scheme 23:**
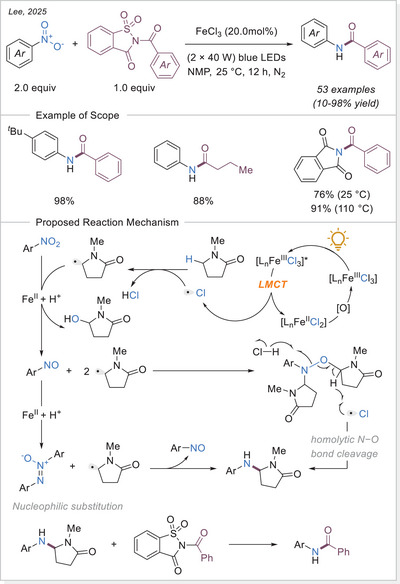
Reductive transamidation of nitroarenes catalyzed by FeCl_3_. NMP, *N*‐methylpyrrolidone.

Lee's group recently developed a sustainable photochemical reductive transamidation of nitroarenes catalyzed by FeCl_3_ under blue LED irradiation (Scheme 23).^[^ ^77^ ^]^ Notably, this study represents the first instance where organic solvents with sacrificial C─H bonds have been employed as reducing agents in transamidation reactions. Among the solvents tested, amide‐based solvents, particularly NMP, delivered the highest yields, with optimized conditions achieving up to 82% of the desired product. Furthermore, the reaction proved effective across various amides with different nitrogen substituents. For instance, 2‐benzoylisoindoline‐1,3‐dione afforded a 76% yield at 25 °C, which significantly increased to 91% when the temperature was raised to 110 °C.

##### C─O bond formation/degradation via HAT

**Scheme 24:**
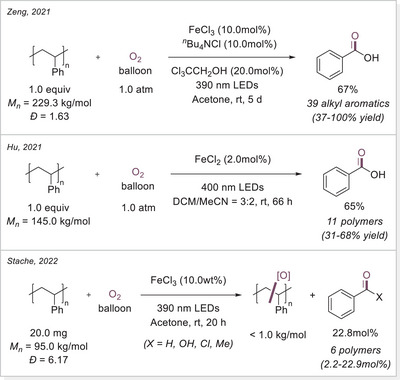
Iron‐photocatalyzed PS degradation strategies via LMCT.

Plastics have become an indispensable part of daily life. However, challenges associated with improper disposal and recycling results in persistent environmental pollution. Therefore, developing efficient methods for chemical plastic recycling under mild and energy‐efficient conditions is an urgent task, redefining plastic waste as a valuable chemical feedstock. In 2021 three independent group published almost at the same time reports on iron‐catalyzed photocatalytic polystyrene (PS) degradation via LMCT (Scheme 24).

Zeng group first explored the degradation of polystyrene (PS) in acetonitrile, achieving benzoic acid in 78% yield under ambient conditions with only 1.0 atm of oxygen pressure (Scheme 24, top).^[^ ^78^ ^]^ Due to the low solubility of PS in acetonitrile, acetone was used as the solvent, and the reaction time was extended. After 5 days, PS underwent C─C bond cleavage, yielding low‐molecular‐weight oligomers and subsequently benzoic acid in 67% yield. Remarkably, the reaction still proceeded efficiently when using direct air exposure instead of an oxygen balloon, affording benzoic acid in 54% yield. Furthermore, real foam samples cut from chemical packaging boxes were successfully degraded to benzoic acid in 64% yield under O_2_.

Hu's group published a related method one month later, for the degradation of plastics, employing FeCl_2_ (2.0mol%) instead of Fe(III) catalysts (Scheme 24, middle).^[^ ^79^ ^]^ To address the solubility issue of PS, a mixed solvent system (DCM/MeCN = 3:2) was used, leading to a 65% yield of benzoic acid after 66 hours, with no detectable oligomers or large molecular fragments. Notably, they demonstrated solvent‐free PS degradation by dissolving PS in FeCl_2_ and then evaporating the solvent, exposing the resulting film to 400 nm LED or natural sunlight. Under these conditions, PS degradation also occurred smoothly, with the molecular weight dropping from 145 to around 50 kg/mol. This discovery suggests potential applications of iron salts as additives for disposable or short‐lifespan plastics.

Stache's Group adopted a similar approach to Zeng's but with simplified additives (Scheme 24, bottom).^[^ ^80^ ^]^ Their study observed an initial increase in benzaldehyde concentration over 12 hours, which later converted predominantly to benzoic acid. They also tested commercial PS samples containing additives like UV absorbers and radical scavengers, which enhance durability. Despite this, PS products from packaging materials to coffee cup lids successfully degraded into PS oligomers. Recognizing the inefficiency of batch reactions in photo‐driven processes, they designed a photo‐flow reactor that enabled the degradation of gram‐scale commercial PS samples within hours, achieving yields comparable to small‐batch reactions. In follow‐up studies, they further demonstrated that bromine radicals could convert PS preferentially into acetophenone rather than benzoic acid.^[^ ^81^ ^]^

**Scheme 25:**
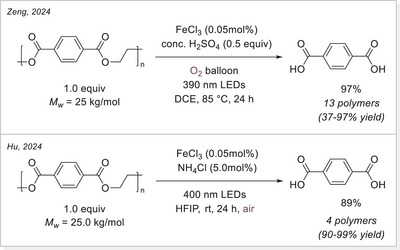
Iron‐photocatalyzed PET degradation strategies via LMCT. HFIP, hexafluoroisopropanol.

Another polymer degradation approach with polyethylene terephthalate (PET) was also investigated concurrently. In 2024, Zeng and Hu independently reported PET photodegradation using the same strategy (Scheme 25).^[^ ^82^ ^]^ Zeng's method involved harsh reaction conditions, employing concentrated H_2_SO_4_ as an additive and heating the reaction mixture to 85 °C. In contrast, Hu used milder conditions, with ammonium chloride as the additive and the reaction conducted at room temperature. Both methods achieved good yields of around 90%.

**Scheme 26:**
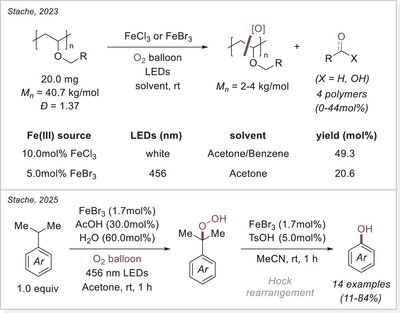
Iron‐photocatalyzed PVE degradation and phenol production via LMCT involving bromine radical.

In 2023, Stache group developed a method for the degradation of polyvinyl ethers (PVEs) using FeCl_3_ or FeBr_3_ as catalyst (Scheme 26, top).^[^ ^83^ ^]^ Product distribution could be tuned by adjusting the reaction conditions. Although bromine radicals are less efficient than chlorine radicals, they allow for higher yields of alcohols and aldehydes.

In 2025, the same group further utilized bromine radicals for phenol production via photooxidation of cumene (Scheme 26, bottom).^[^ ^84^ ^]^ The intermediate, cumene hydroperoxide (CHP), could be stabilized by the addition of acetic acid and water, leading to increased yield of phenol. These studies collectively highlight the potential of iron‐mediated LMCT photocatalysis as a promising approach for sustainable plastic degradation and recycling.

**Scheme 27:**
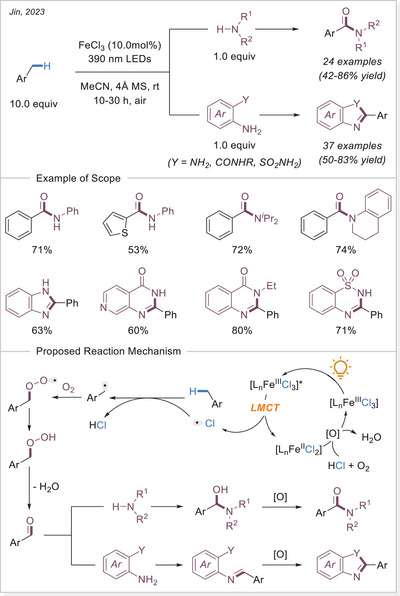
Fe‐catalyzed benzylic C─H oxidative amidation.

Beyond the oxidation of C(sp^3^)─H bonds, further transformations could also be achieved, enabling a one‐pot amidation of the resulting aldehyde. In 2023, Jin's group reported a photoinduced iron to ligand charge transfer strategy for benzylic C─H oxidative amidation reactions (Scheme 27).^[^ ^85^ ^]^ This protocol is broadly applicable, accommodating not only simple alkyl and aromatic amines but also a diverse range of *ortho*‐amino arylamides and *ortho*‐aminoanilines.

##### C─S, C─Se, and C─B bond formation via HAT

**Scheme 28:**
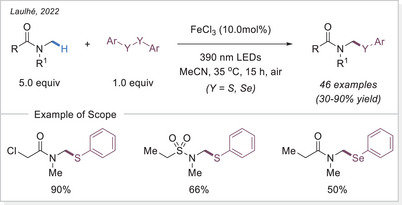
Light‐induced iron‐catalyzed C─H sulfinylation and selenylation.

Sulfur‐containing compounds comprise more than 20% of FDA‐approved drugs, ranking sulfur as the third most common heteroatom in pharmaceuticals after oxygen and nitrogen.^[^ ^86^ ^]^ Direct C─H thiolation of amides presents a highly appealing approach for late‐stage functionalization and structural diversification, as it circumvents the use of unstable intermediates. In 2022, Laulhé’s group reported a photoinduced C─H functionalization of amides and ethers, enabling the formation of C─S and C─Se bonds (Scheme 28).^[^ ^87^ ^]^ This protocol exhibits broad functional group compatibility with synthetically valuable moieties, including Boc‐ and mesyl‐protected amines, nitriles, halogens, and sulphonamides, which are particularly relevant to medicinal chemistry.

**Scheme 29:**
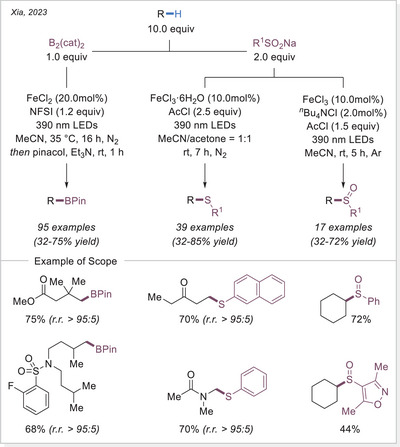
C─H sulfinylation, sulfonylation, and borylation. NFSI, *N*‐fluorobenzenesulfonimide.

Direct C─H sulfinylation, along with sulfonylation, and borylation, can be efficiently achieved via an iron‐mediated photoreaction.^[^ ^88^ ^]^ All three reactions leverage the photoinduced Fe‐LMCT strategy, reported by Xia's group, demonstrating high regioselectivity for stronger primary C─H bonds over tertiary C─H bonds, which differs significantly from conventional chlorine radical‐mediated HAT protocols (Scheme 29). Notably, the substrate scope extends beyond simple alkanes to a diverse range of C(sp^3^)─H‐containing molecules, including ketones, nitriles, esters, ethers, amides, sulfonamides, halides, and silanes.

**Scheme 30:**
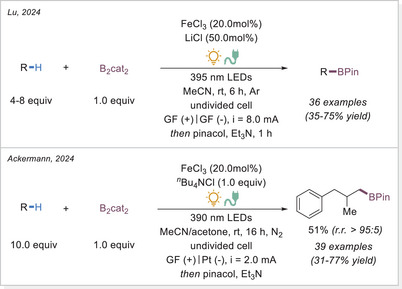
Photoelectrochemical iron‐catalyzed C(sp^3^)─H borylation.

In 2024, Lu and Ackermann independently reported photoelectrochemical C─H borylation methods (Scheme 30).^[^ ^89^ ^]^ Both used FeCl_3_ as the catalyst, and a variety of substrates containing multiple hydridic C─H bonds selectively yielded borylated products at the primary C(sp^3^)─H positions, overriding the typically more reactive tertiary or benzylic sites. Ackermann's study proposed that this unusual selectivity for primary C─H bonds may be due to the ease of overoxidation of benzylic and α‐heteroatom carbon‐centered radicals. Interestingly, despite the challenge of over‐functionalization, Lu's group also investigated methane as a substrate and achieved 18% yield under high pressure (5.5 MPa), as detected by GC.

**Scheme 31:**
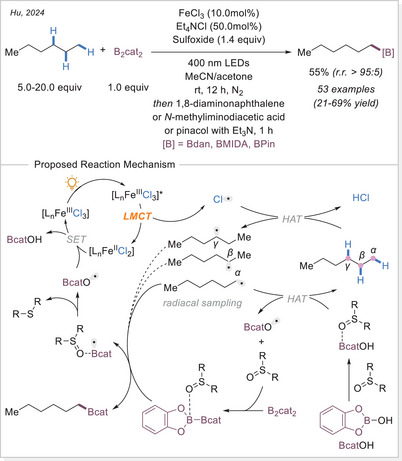
Fe‐catalyzed selective borylation of terminal C(sp^3^)─H bonds.

In 2024, Hu's group reported an iron‐catalyzed selective borylation of unbranched alkanes with varying degrees of steric hindrance (Scheme 31).^[^ ^90^ ^]^ The regioselectivity is proposed to be directed by an in‐situ generated boron–sulfoxide complex. Using diphenyl sulfoxide, borylation selectively occurred at sterically accessible terminal C─H bonds, while secondary positions were completely unreactive. This strategy enables the selective functionalization of C(sp^3^)─H bonds over C(sp^2^)─H and C(sp)─H bonds. For substrates without regioselectivity challenges or those bearing steric hindrance or electron‐withdrawing groups, DMSO proved to be an effective oxidant. Notably, both branched alkanes and silanes underwent selective borylation under these conditions.

##### C─P bond formation via HAT

Organophosphines are of crucial importance in a number of fields of inquiry, including chemistry, materials science, and life sciences, with widespread applications in both academia and industry. Among them, tertiary phosphines P(III) have been recognized as key reactants, organocatalysts, and ligands in numerous synthetically significant transformations as well as in sophisticated asymmetric synthesis.

**Scheme 32:**
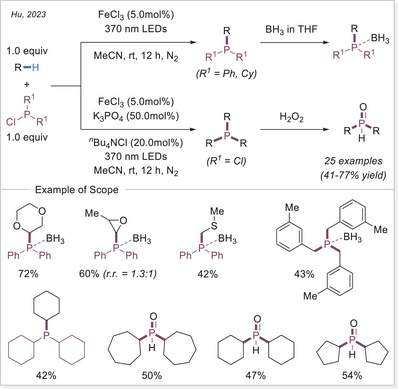
Iron‐catalyzed C(sp^3^)─H phosphorylation.

In 2023, Hu's group reported C(sp^3^)─H phosphorylation via an FeCl_3_‐mediated LMCT process, enabling the synthesis of structurally diverse tertiary phosphines (Scheme 32).^[^ ^91^ ^]^ Given the air sensitivity of the resulting tertiary phosphines, they were treated with borane post‐reaction, allowing for the facile isolation of their corresponding phosphine‐borane complexes. Notably, the scope of phosphine sources is broad, including diphenylphosphine chloride, dicyclohexylchlorophosphine, trichlorophosphine, which proved to be effective reagents. The reactions with PCl_3_ yielded dialkyl substituted phosphines, which, upon H_2_O_2_ workup, furnished phosphine oxides in good yields. Interestingly, benzylic C─H bonds also exhibited preferential reactivity with PCl_3_, affording tertiary phosphines.

**Scheme 33:**
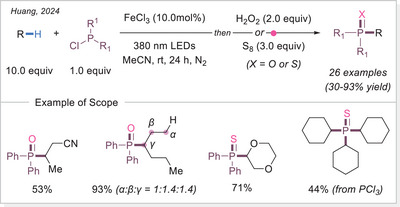
Iron‐catalyzed C(sp^3^)─H phosphorylation for the synthesis of phosphine oxides and sulfides.

In 2024 Huang's group employed a similar Fe‐LMCT radical system for C─H phosphorylation using chlorophosphines, yielding phosphine oxides after H_2_O_2_ treatment (Scheme 33).^[^ ^92^ ^]^ Additionally, they broadened the substrate scope to phosphine sulfide by using elemental sulphur (S_8_) instead of H_2_O_2_ as the quenching agent.

##### C─halogen bond formation via HAT

**Scheme 34:**
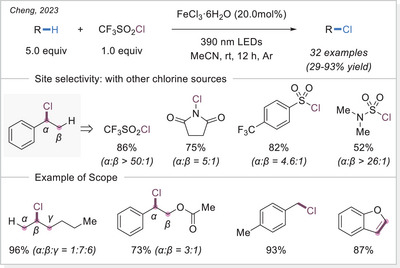
Ligh‐induced iron‐catalyzed highly regioselective benzylic C─H chlorination.

In 2023, Cheng's group developed a highly regioselective method for benzylic C─H chlorination (Scheme 34).^[^ ^93^ ^]^ Various chlorinating reagents were evaluated, with trifluoromethanesulfonyl chloride emerging as the most effective, achieving an excellent yield of 95% and exceptional site selectivity (α:β > 50:1). Furthermore, the reaction demonstrated broad substrate compatibility, extending to compounds lacking benzylic C─H bonds. Interestingly, in the case of 2,3‐dihydrobenzofuran, the formation of alkenes was favored over direct chlorination.

#### 4.1.2 Substrate Activation via C─C, X─H, C─X, and X─X Bond Cleavage by Halo Radicals Generation

##### C─Se bond formation via Halo radicals generation

**Scheme 35:**
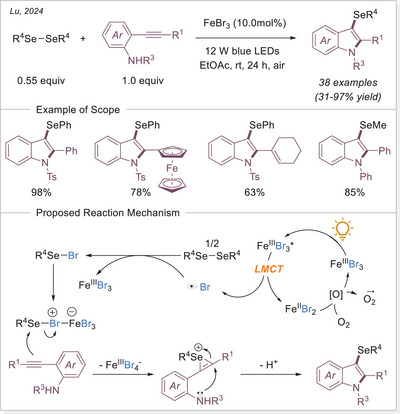
Bromide radical‐mediated selenocyclization under iron catalysis.

As stated at the beginning of this chapter, Fe(III)─Br complexes can also serve as effective radical generators. Using conditions similar to those reported by Xia,^[^ ^94^ ^]^ in 2024, Lu's group introduced a method for the selenocyclization of 2‐ethynylanilines with diselenides, utilizing FeBr_3_ as both a photoinduced LMCT catalyst and a Lewis acid (Scheme 35).^[^ ^95^ ^]^ Notably, substituting FeBr_3_ with FeCl_3_ led to a significant drop in yield from 95% to 22%, highlighting the critical role of bromide ligands in the catalytic process. This approach provides an efficient strategy for synthesizing a wide range of 3‐selenylindoles with diverse substitution patterns.

##### C─halogen bond formation via Halo radicals generation

**Scheme 36:**
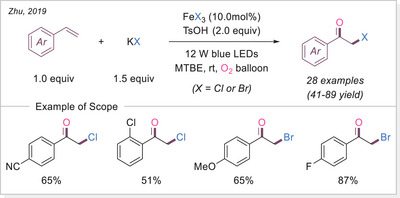
Synthesis for α‐haloketones via LMCT‐induced iron catalysis. MTBE, methyl‐*tert*‐butylether.

Zhu's group established an iron‐catalyzed approach for synthesizing α‐haloketones under light irradiation (Scheme 36).^[^ ^96^ ^]^ The reaction successfully utilizes both bromide and chloride radicals. While their proposed mechanism does not explicitly mention the LMCT process, it is likely involved in facilitating the transformation.

**Scheme 37:**
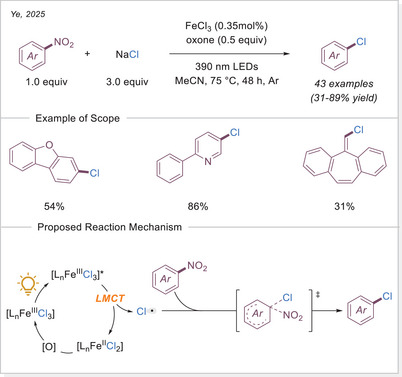
Light‐promoted iron‐mediated denitrative chlorination strategy for aryl and heteroaryl compounds.

Recently, Ye's group reported a denitrative chlorination strategy for aromatic rings (Scheme 37).^[^ ^97^ ^]^ In this method, chlorine radical facilitates a radical substitution reaction, cleaving the C_Ar_─NO_2_ bond under mild conditions while releasing NO_2_ gas, converting nitroarenes into the corresponding aryl chlorides. This practical approach is compatible with a broad range of unactivated nitro(hetero)arenes and nitroalkenes and operates efficiently without sensitivity to air or moisture.

##### C─Si and C─Ge bond formation via halo radicals generation

**Scheme 38:**
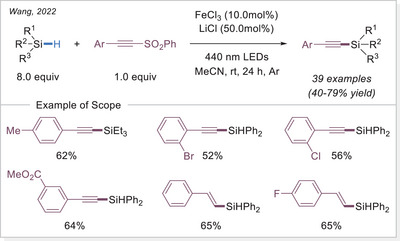
Iron‐photocatalyzed Si─H alkynylation via LMCT.

Not only carbon alkanes but also hydrosilanes can serve as substrates for the HAT process via the iron‐catalyzed LMCT mechanism. Similar to Duan's research discussed in Section 3.1.1, Wang's group developed an alkynylation strategy using phenyl sulfone as the alkynylation reagent, but instead of alkanes, they employed hydrosilanes to generate value‐added silicon compounds (Scheme 38).^[^ ^98^ ^]^ The chloride radical generated from the LMCT process was capable of abstracting a hydrogen atom from the Si─H bond, leading to the formation of a silyl radical. The reaction exhibited broad substrate compatibility, accommodating alkynes with various functional groups and steric properties, as well as both alkyl and aryl hydrosilanes. Notably, the reaction of vinyl sulfones selectively yielded *E‐*vinylsilanes, offering a potential solution to the long‐standing challenge of regio‐ and stereoselective vinyl silane synthesis.

**Scheme 39:**
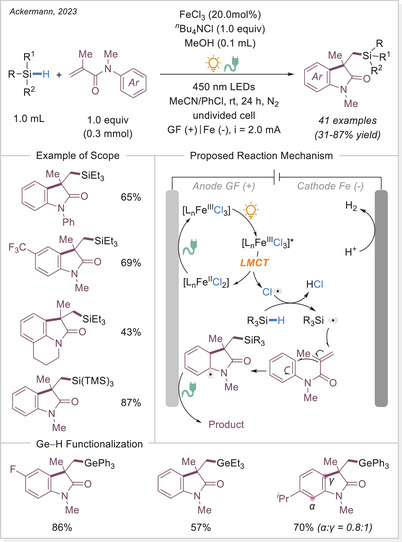
Iron‐catalyzed LMCT‐induced electrocatalysis strategy for silane and germane activation. GF, graphite felt.

In 2023, Ackermann's group reported an iron‐catalyzed LMCT strategy combined with electrocatalysis for hydrosilane activation, enabling the formation of C─Si bonds (Scheme 39).^[^ ^99^ ^]^ The generated Cl radical undergoes a radical‐polarity‐matched HAT process, selectively forming a silyl radical. This is followed by radical addition, anodic oxidation, and deprotonation to afford the desired product. Meanwhile, methanol is reduced at the cathode, producing hydrogen gas. Notably, this method could activate not only Si─H bonds but also Ge─H bonds. Organogermanes, known for their high hydrophobicity and low toxicity, have recently emerged as alternative and orthogonal coupling partners in synthesis and catalysis. To further explore their synthetic potential, the researchers expanded the substrate scope to include germanium‐based functionalities. Both triphenyl and trialkyl germanes were demonstrated to be suitable substrates for Ge incorporation, highlighting the versatility of this approach.

#### 4.1.3 C─C Bond Cleavage of Alcohols via Proton‐Coupled Electron Transfer (PCET)

Deconstructive strategies involving C─C bond cleavage represent a powerful synthetic logic, unlocking diverse pathways for molecular editing and enabling rapid access to new scaffolds. However, the selective activation of inert C─C bonds remains a challenge due to their high bond dissociation energies, often necessitating the use of pre‐activated or strained substrates. Recent advancements have focused on heterolytic C─C bond cleavage, though these approaches are typically restricted to strained ring systems.^[^ ^100^ ^]^ Alternatively, homolytic C─C bond scission has emerged as a promising tactic to overcome these substrate constraints, offering new opportunities for broadening molecular diversity through radical‐mediated transformations. In this context, iron‐catalyzed LMCT‐mediated homolytic C─C bond cleavage of alcohol through PCET^[^ ^101^ ^]^ has gained significant attention as an efficient and mild method to generate alkyl radicals from otherwise inert substrates. In the following sections, we summarize recent developments that employ this LMCT‐driven approach, highlighting its growing impact on selective C─C bond cleavage and functionalization.^[^ ^102^ ^]^

**Scheme 40:**
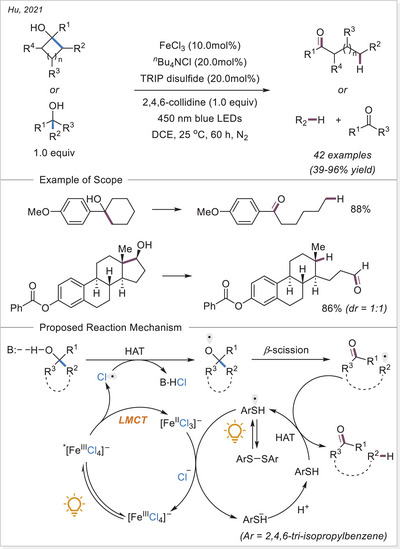
Iron‐catalyzed LMCT‐mediated C─C single‐bond cleavage of alcohols. B, base; TRIP, 2,4,6‐triisopropylphenyl.

In 2021, the Hu group developed an iron‐photocatalyzed strategy for deconstructive C─C bond cleavage of alcohols under visible light irradiation, enabling the transformation of primary, secondary, and tertiary alcohols (both cyclic and linear) into ketones or aldehydes (Scheme 40).^[^ ^103^ ^]^ The method leverages LMCT excitation of an iron(III)‐chloride complex to generate chlorine radicals, which initiate the reaction by abstracting a hydrogen atom from the alcohol's O─H bond via a PCET mechanism, assisted by 2,4,6‐collidine as base. The resulting alkoxy radicals undergo β‐scission to cleave adjacent C─C bonds, forming carbon‐centered radicals and carbonyl products. Importantly, disulfides are employed as HAT reagents; their homolytic cleavage furnishes thiyl radicals that efficiently quench the transient carbon‐centered radicals, thus preventing undesired radical‐radical coupling. This strategy showcases a mild, operationally simple protocol with broad substrate scope, highlighting the potential of iron‐LMCT photocatalysis combined with disulfide‐mediated HAT in sustainable molecular editing via C─C bond cleavage.

**Scheme 41:**
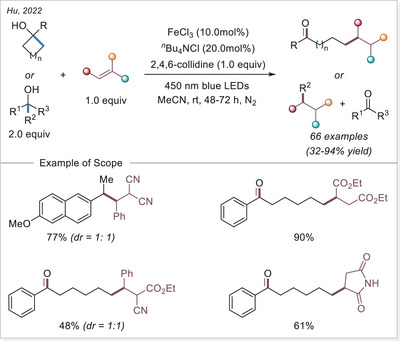
Iron‐catalyzed LMCT‐induced deconstructive alkylation through C─C bond cleavage of alcohols.

In 2022, the same research group extended this methodology to achieve alkene hydrofunctionalization through a deconstructive alkylation strategy (Scheme 41).^[^ ^104^ ^]^ In this approach, the generated chlorine radicals from FeCl_3_ abstract a hydrogen atom from alcohols via PCET mechanism, forming reactive alkoxy radicals. These alkoxy radicals undergo β‐scission to cleave adjacent C─C bonds, yielding carbon‐centered radicals. The resulting radicals then engage in intermolecular addition to electron‐deficient alkenes, forging new C─C bonds.

**Scheme 42:**
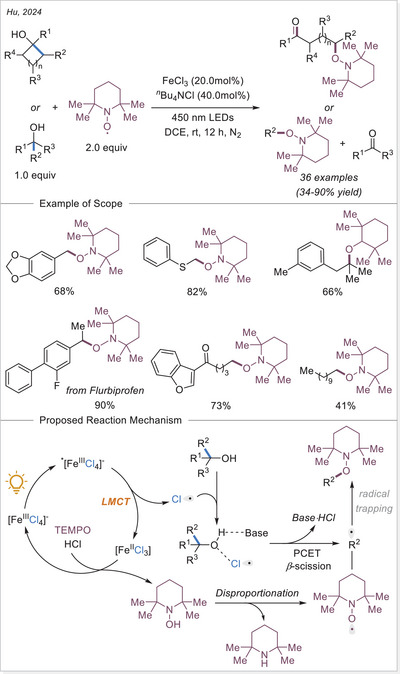
Iron‐catalyzed alkoxyamination through C − C bond cleavage of alcohols.

In 2023, the same group developed another ironcatalyzed deconstructive cyanomethylation strategy that transforms both cyclic and linear alcohols into cyanomethylated products via radical crosscoupling with acetonitrile.^[^ ^105^ ^]^ In next year, Hu group reported a protocol where alkyl alcohols transformed into alkoxyamination products via deconstructive C─C bond cleavage and paired with C─O bond formation under mild conditions (Scheme 42).^[^ ^106^ ^]^ The mechanism begins with LMCT excitation of an ironchloride complex, generating chlorine radicals that abstract hydrogen from the alcohol via PCET, yielding alkoxy radicals. Although two possible pathways were proposed based on the mode of alkoxy radical generation, we present here the most likely mechanism. After β‐scission, the resulted carbon‐centered radicals are trapped by oxygen centered nucleophiles (TEMPO derivatives), forging new C─O bonds. Notably, the addition of *^t^* Bu_4_NCl promotes the formation of [FeCl_4_]^−^ complexes instead of TEMPO‐coordinated iron species, thereby suppressing the oxidation pathway of hydroxyl groups, enabling compatibility with primary and secondary alcohols that are typically prone to overoxidation. The methodology exhibits broad substrate scope, late‐stage functionalization, and post‐synthetic modification, providing a practical platform for deconstructive alkoxyamination of otherwise inert C─C bonds.

### 4.2 LMCT of Carboxylate‐Iron Species in Organic Synthesis

The decarboxylation of carboxylic acids has historically served as a cornerstone transformation in organic synthesis, enabling the construction of both carbon─carbon and carbon─heteroatom bonds. Despite their utility, conventional decarboxylation strategies often suffer from significant drawbacks, including the need for pre‐activation of the acid, reliance on expensive or toxic heavy metal catalysts, and harsh thermal conditions.^[^ ^107^, ^125^ ^]^ Recent advances in photochemistry have transformed this landscape, introducing light‐driven decarboxylation as a milder and more sustainable alternative. In particular, photocatalytic systems employing first‐row transition metals; such as iron, copper, and cerium, have garnered growing interest.^[^ ^24^ ^]^ These metals form photoactive carboxylate complexes that, upon irradiation, undergo excitation and subsequent homolytic cleavage of the metal─oxygen bond, liberating carbon dioxide and generating reactive intermediates. Crucially, these systems leverage inner‐sphere electron transfer mechanisms, circumventing the need for long‐lived excited states. This not only enhances site‐selectivity but also reduces undesired side reactions, positioning these methods as highly selective and environmentally friendly. In this review, the integration of inexpensive, earth‐abundant iron metals into photochemical decarboxylation platforms highlights a promising direction for greener, more efficient synthetic methodologies. This section explores how light‐driven strategies can transform particularly electron‐deficient and alkyl carboxylic acids into reactive radical species, through SET oxidation and LMCT pathways.^[^ ^108^ ^]^ Emphasis is placed on their application in the late‐stage modification of structurally complex molecules, highlighting how decarboxylation can be harnessed for selective functionalization. Alongside these transformations, the section also delves into the underlying mechanistic principles that drive these radical‐generating processes.

#### 4.2.1 Decarboxylative C─C Bond Formation

##### Minisci‐type reactions

**Scheme 43:**
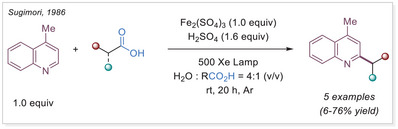
Pioneering example of LMCT‐enabled decarboxylative Minisci‐type alkylation of heteroarenes.

**Scheme 44:**
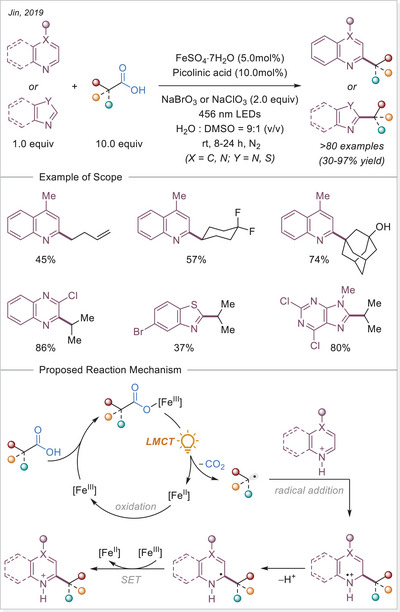
Iron(III)‐catalyzed decarboxylative Minisci‐type alkylation of heteroarenes.

A pioneering example of LMCT‐enabled decarboxylation was reported by Sugimori in 1986, demonstrating iron‐promoted C─H alkylation of quinolines via decarboxylation of alkyl carboxylic acids under light irradiation (Scheme 43).^[^ ^22^, ^109^ ^]^ This early study utilized stoichiometric Fe(III) salt and large excess of the acid as a co‐solvent, marking the first synthetic application of iron‐mediated LMCT decarboxylation via a Minisci‐type mechanism. Decades later, Jin group advanced this concept into a catalytic process using blue light and terminal oxidants such as NaBrO_3_ or NaClO_4_ to regenerate Fe(III) in 2019 (Scheme 44).^[^ ^23^ ^]^ The inclusion of picolinic acid as a ligand proved to be crucial as the yield increased dramatically upon its addition. The reaction exhibited broad functional group tolerance across a wide range of both carboxylic acid and heteroarene substrates, establishing a versatile platform for Minisci‐type alkylations, though still reliant on high acid loading.

**Scheme 45:**
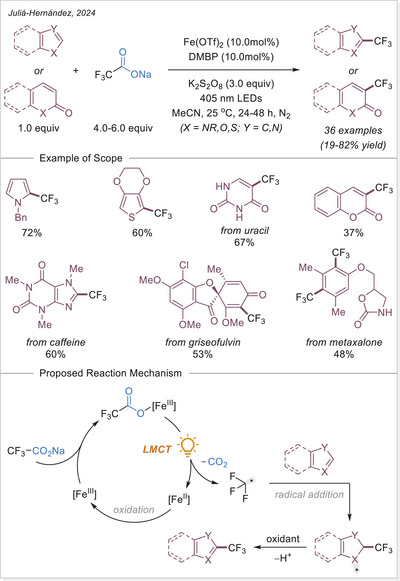
Fe‐catalyzed trifluoromethylation of (hetero)arenes using trifluoroacetates. DMBP, 4,4′‐dimethoxy‐2,2′‐bipyridine.

Afterwards, in 2024, Juliá‐Hernández and coworkers introduced an iron‐catalyzed, photoinduced LMCT strategy that effectively addresses the challenges associated with the high oxidation potential of trifluoroacetate salts in decarboxylation reactions (Scheme 45).^[^ ^110^ ^]^ This innovative approach prevents the redox degradation of trifluoroacetate during decarboxylation, thereby broadening the applicability of LMCT photocatalysis. The active catalyst is generated in‐situ by coordinating the ligand with Fe(OTf)_2_, which then binds to sodium trifluoroacetate. Upon visible light irradiation, homolytic cleavage of the Fe─O bond occurs, leading to decarboxylation and formation of the trifluoromethyl radical. This radical subsequently reacts with various electron‐rich (hetero)aromatics and complex molecules, undergoing oxidation and aromatization steps to yield trifluoromethylated products. This method enables efficient introduction of trifluoromethyl groups into a wide range of substrates, with late‐stage functionalization highlighting the significant potential of photodecarboxylation in synthetic chemistry.

**Scheme 46:**
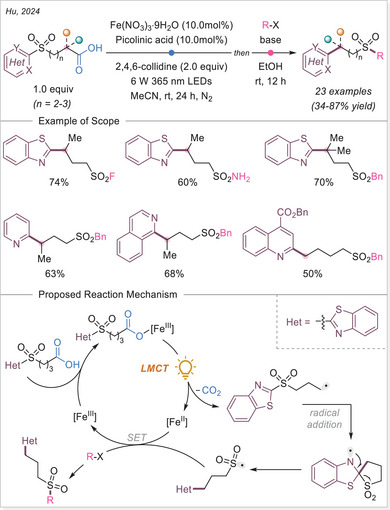
Fe‐catalyzed SO_2_ retaining radical Smiles rearrangement reaction through decarboxylation. Het, heteroarene.

Recent advancements in iron‐catalyzed photochemical transformations have expanded the scope of decarboxylative processes, particularly in the context of the SO_2_‐retaining Smiles rearrangement. Hu introduced a novel iron‐catalyzed method that utilizes visible light to facilitate the decarboxylation of aliphatic carboxylic acids in 2024 (Scheme 46).^[^ ^111^ ^]^ The key to this process is the in‐situ generation of an active Fe(III) catalyst, which, upon light activation, undergoes homolytic cleavage, forming alkyl radicals.

These reactive species subsequently add to electron‐deficient π‐systems, leading to the formation of sulfonylated products. This strategy overcomes previous limitations posed by high oxidation potentials and demonstrates the power of LMCT photocatalysis in driving efficient and selective organic transformations, highlighting its broad potential in synthetic chemistry.

**Scheme 47:**
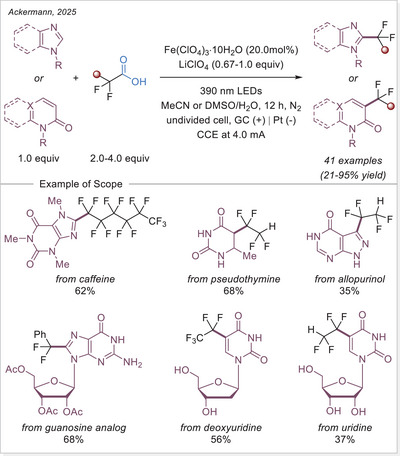
Photoelectrochemical iron(III) catalysis for late‐stage C─H fluoroalkylations. CCE, constant current electrolysis, GC, glassy carbon.

This year, Ackermann group developed photoelectrocatalytic iron(III) system that combines anodic oxidation with photoexcitation to achieve late‐stage C─H fluoroalkylation of biologically relevant heterocycles (Scheme 47).^[^ ^112^ ^]^ Leveraging earth‐abundant iron catalysts, the method enables direct incorporation of fluoroalkyl radicals into xanthines, nucleobases, and nucleosides type bioactive molecule, while concurrently driving hydrogen evolution. Mechanistic studies reveal that LMCT triggers fluoroalkyl radical formation, supporting the chemo‐ and site‐selectivity of this sustainable strategy. Overall, the work highlights the powerful synergy between photochemical and electrochemical approaches, providing a scalable and sustainable strategy for selective C─H functionalization.

##### Giese‐type addition

**Scheme 48:**
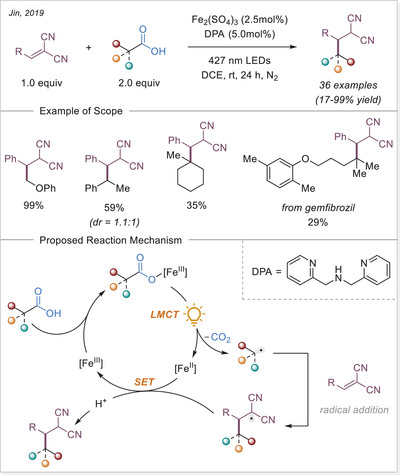
Fe‐catalyzed decarboxylative Giese‐type reaction with Michael acceptors. DPA, di(2‐picolyl)amine.

Expanding the utility of LMCT‐based decarboxylative strategies, the Jin group subsequently developed an iron‐catalyzed platform for alkylation and amination reactions involving alkyl carboxylic acids (Scheme 48).^[^ ^23^ ^]^ In this system, photoexcited Fe(III)─carboxylate complexes undergo LMCT to generate alkyl radicals, which add to electron‐deficient π‐systems such as olefins possessing two electron withdrawing group or azodicarboxylates. Remarkably, the resulting electrophilic radical intermediates facilitate catalyst turnover by oxidizing Fe(II) back to Fe(III), eliminating the need for an external oxidant. Ligand effects were again pronounced, with 2,2′‐picolylamine proving essential for high efficiency. Notably, other transition metals including Mn, Ni, Co, and Cu, failed to mediate the transformation under analogous conditions.

The reaction displayed broad compatibility with a wide range of alkyl carboxylic acids, including primary, secondary, tertiary, benzylic, and α‐heteroatom‐substituted variants. Although the scope of olefinic partners was more limited, particularly to malononitrile derivatives, the overall transformation offered a versatile and oxidant‐free route to C─C and C─N bond formation. In the amination protocol, azodicarboxylates served as radical acceptors, with excess carboxylic acid required to drive the transformation.

**Scheme 49:**
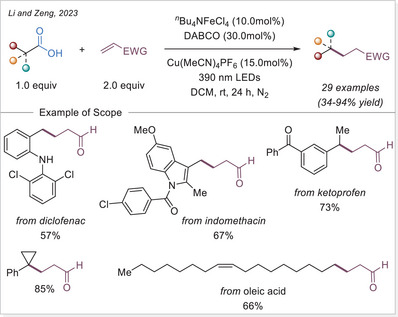
Decarboxylative Giese‐type reaction with electron‐deficient alkenes through a dual iron and copper catalytic system. DABCO, 1,4‐diazabicyclo[2.2.2]octane.

A dual catalytic system combining copper and iron catalysis has been demonstrated to efficiently promote decarboxylative alkylation reactions, particularly with electron‐deficient alkenes by Li and Zeng in 2023 (Scheme 49).^[^ ^113^ ^]^ The iron catalyst *^n^* Bu_4_NFeCl_4_ alone yielded the desired product in only 45%, but upon introducing Cu(MeCN)_4_PF_6_ as a co‐catalyst, the yield increased to 90%. The process is initiated by an LMCT mechanism, where the iron catalyst undergoes visible light‐induced excitation, leading to the homolytic cleavage of the Fe─O bond and generating reactive alkyl radicals. These radicals then participate in a Giese‐type reaction, where they add to the electron‐deficient alkenes, such as acrolein, to form new C─C bonds. The copper catalyst might act as a Lewis acid, facilitating the activation of the electron‐deficient olefins and inhibiting potential polymerization. This dual catalytic approach exhibited impressive versatility, enabling the alkylation of a wide range of carboxylic acids, including primary, secondary, and tertiary acids, as well as various natural products and pharmaceuticals. This highlights the method's potential for post‐modification of complex molecules in synthetic chemistry.

**Scheme 50:**
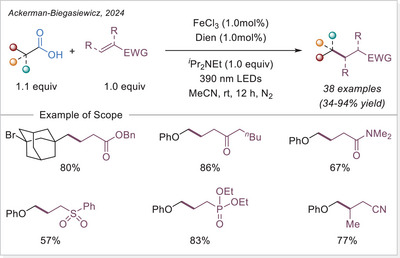
Iron photodecarboxylative Giese‐type addition to Michael acceptors. Dien, diethylenetriamine.

In the same year, Ackerman‐Biegasiewicz and coworkers reported a benchtop‐stable iron diethylenetriamine system for decarboxylative Giese‐type additions to activated alkenes (Scheme 50).^[^ ^53^ ^]^ This Fe‐based photocatalyst formed in‐situ, tolerates ambient conditions and avoids moisture sensitivity which is common in many Fe systems. Under blue light, the complex undergoes LMCT‐activated decarboxylation, generating carbon radicals that add efficiently to electron‐deficient olefins (acrylates, vinyl ketones, acrylonitrile). The authors demonstrate broad acid compatibility, including electron‐rich amines and amino acids, with high yields (38–90%). Combined kinetic, mechanistic, and DFT studies confirm radical‐polar crossover reactivity with iron facilitating both radical formation and subsequent electron transfers. This work underscores how ligand design enhances catalyst robustness, broadening Giese‐type radical chemistry under iron photocatalysis. For a detailed proposed reaction mechanism, please see Section 3.1 (Scheme 7) of this review.

**Scheme 51:**
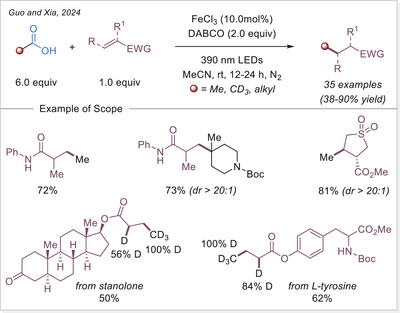
Fe photodecarboxylative hydromethylation of alkenes. DABCO, 1,4‐diazabicyclo[2.2.2]octane.

In the same year, Guo and Xia reported another highly significant methodology involving photoinduced hydromethylation of alkenes enabled by LMCT. In this strategy, methyl or CD_3_ radicals, traditionally challenging to generate, are formed via decarboxylation of acetic or deuterated acetic acid (Scheme 51).^[^ ^114^ ^]^ These radicals undergo efficient addition to a broad range of alkenes under mild conditions. The selective incorporation of methyl or deuteromethyl groups is particularly valuable and in high demand in medicinal chemistry, as such modifications can profoundly impact biological activity and metabolic stability.

**Scheme 52:**
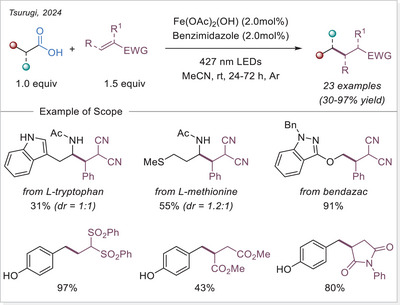
Decarboxylative alkylation of carboxylic acids catalyzed by an imidazole‐coordinated Fe_3_‐cluster under visible light irradiation.

Tsurugi group reported a visible‐light‐driven decarboxylative alkylation protocol that couples a broad range of carboxylic acids including those bearing easily oxidizable phenol and indole moieties with alkenes (Scheme 52).^[^ ^57^ ^]^ The reaction is catalyzed by in‐situ generated imidazole‐coordinated Fe_3_(μ‐O) cluster ([Fe(OAc)_2_(OH)] and benzimidazole), which displays enhanced visible‐light absorption and milder oxidative power. Kinetic studies reveal first‐order dependence on alkene concentration and linear correlation with light intensity, indicating LMCT initiation from the Fe cluster. Importantly, the mild redox potential of the Fe_3_‐cluster enables tolerance of sensitive functionalities that are usually incompatible with conventional Fe(III) systems. Mechanistically, LMCT from Fe–carboxylate complexes drive decarboxylation to form alkyl radicals, which undergo radical addition to alkenes, yielding alkylated products in good to excellent yields. This work highlights a novel strategy to expand the substrate scope of iron‐photocatalyzed decarboxylative functionalizations to oxidation‐sensitive substrates through cluster engineering. For a detailed proposed reaction mechanism, please see Section 3.3 (Scheme 9) of this review.

**Scheme 53:**
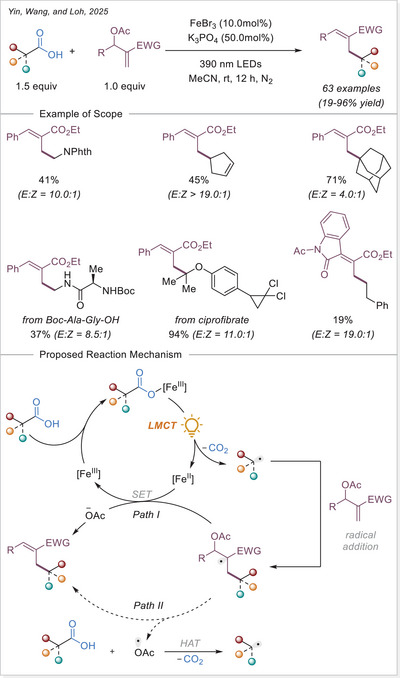
Iron‐photocatalyzed decarboxylative alkylation of carboxylic acids with Morita − Baylis − Hillman acetates.

Very recently in 2025, Yin, Wang, and Loh developed an iron‐photocatalyzed coupling between carboxylic acids and Morita − Baylis − Hillman (MBH) acetates for stereoselective construction of *E*‐configured tri‐ and tetrasubstituted alkenes (Scheme 53).^[^ ^115^ ^]^ Under visible‐light LMCT conditions with Fe(II)/Lewis base catalytic system, a radical from the acid adds to the MBH acetate, expelling the leaving group and delivering densely substituted alkenes with high *E*/*Z* control (up to > 19:1). Over 60 diverse examples, including tertiary acids and pharmaceutical derivatives, were achieved in good to excellent yields (up to 96%). Mechanistic probes suggest a radical‐polar crossover mechanism following LMCT decarboxylation, providing a powerful tool for alkene construction from carboxylic acids.

##### Hydrofunctionalization of alkenes

**Scheme 54:**
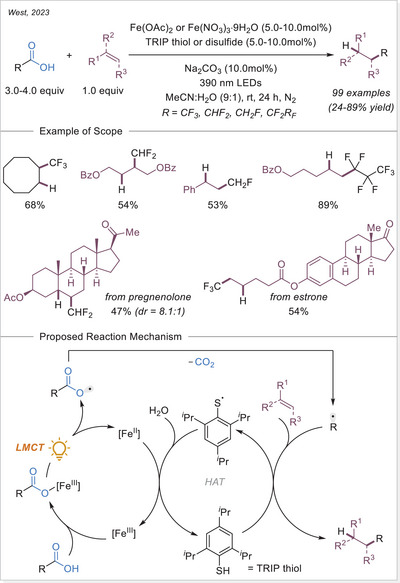
Iron‐photocatalyzed hydrofluoroalkylation of alkenes with carboxylic acids. TRIP, 2,4,6‐triisopropylphenyl.

West group reported an iron/thiol dual‐catalytic hydrofluoroalkylation method that directly employs fluoroalkyl carboxylic acids (from mono‐ to perfluoroalkyl) and alkenes under visible light without any preactivation or noble metals requirement (Scheme 54).^[^ ^116^ ^]^ LMCT from C_n_F_m_–COO‐Fe(III) complexes generates fluoroalkyl radicals, which add to alkenes, followed by thiol‐mediated HAT to afford hydrofluoroalkylated products. This strategy effectively overcomes the high oxidation potential of fluoroalkyl acids and demonstrates broad substrate scope, including bioactive and terminal/internal alkenes. Mechanistic experiments support radical generation through Fe‐based LMCT and radical hydrogen transfer with a thiol co‐catalyst, highlighting modularity and practicality in the synthesis of pharmaceutically relevant motifs.

**Scheme 55:**
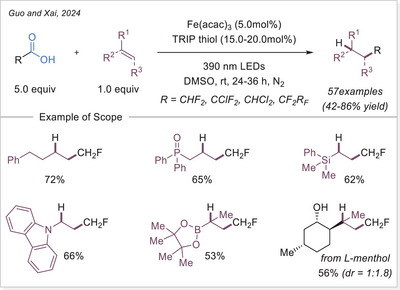
Iron‐photocatalyzed decarboxylative hydrodifluoromethylation and hydromethylation of alkenes. TRIP, 2,4,6‐triisopropylphenyl.

In 2024, Guo and Xia group developed an innovative iron‐catalyzed LMCT decarboxylation strategy that effectively activates various inert haloalkyl carboxylates (C_n_X_m_COO−, X = F or Cl) using a HAT reagent (Scheme 55).^[^ ^114^ ^]^ Upon visible light irradiation, the iron complex undergoes LMCT to produce a trifluoroacetic acid radical, which rapidly decarboxylates to yield a trifluoromethyl‐type (C_n_X_m_•) radical. This radical then reacts with olefins to form a radical adduct, which subsequently undergoes HAT process to produce the desired product. Notably, this method demonstrates broad substrate applicability, effectively accommodating various aliphatic terminal alkenes containing benzoyl groups in the presence of strong Brønsted acids.

In a recent report, West and Katayev coworkers reported the first efficient hydroalkylation of alkenes using highly reactive CF_3_‐substituted cyclopropyl and cyclobutyl radicals, generated via LMCT‐excited iron‐catalyzed decarboxylation process.^[^ ^117^ ^]^ This study not only addresses the long‐standing challenge of decarboxylating CF_3_‐containing strained rings but also offers deep mechanistic insight through complementary experimental, spectroscopic, and DFT studies, supported by a radical stabilization energy (RSE) framework.

##### 1,2‐difunctionalization of alkenes

**Scheme 56:**
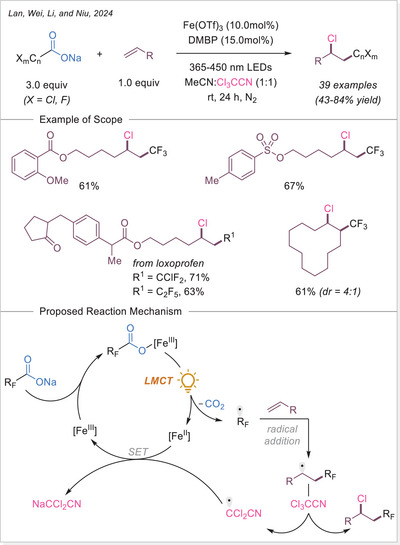
Iron‐photocatalyzed decarboxylative chloro/fluoro‐polyhaloalkylation of alkenes. DMBP, 4,4′‐dimethoxy‐2,2′‐bipyridine.

In 2024, Niu and coworkers developed a dualligand iron photocatalytic system for the activation of haloalkyl carboxylates (C_n_X_m_–COO^−^, X = F, Cl, Br) via LMCT, yielding C_n_X_m_ radicals for selective polyhaloalkylation of unactivated alkenes (Scheme 56).^[^ ^118^ ^]^ Using bipyridine and acetonitrile/trichloroacetonitrile as complementary ligands, they assemble potent lightharvesting Fe‐haloalkyl carboxylate complexes. The protocol enables both chloro‐ and fluoro‐polyhaloalkylation in moderate to excellent yields, is effective in late‐stage modifications, and features low iron loading (TON ≈ 257) and gram‐scale capability. Mechanistic investigations, including UV‐Vis and control experiments, show that ligand combination enhances LMCT and radical release, enabling sustainable transformation of bulk halogen‐containing acids under visible light.

**Scheme 57:**
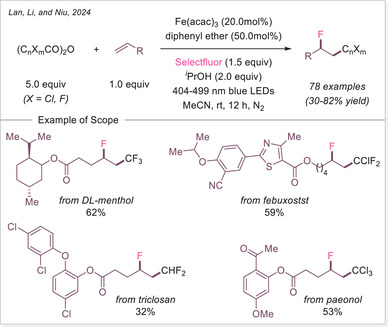
Iron‐photocatalyzed decarboxylative fluoro‐trifluoromethylation of alkenes.

In the same year, Niu and colleagues again revealed that adding a Brønsted acid dramatically enhances LMCT activation of inert haloalkyl carboxylates (e.g., CF_3_COO^−^) under visible light (Scheme 57).^[^ ^119^ ^]^ The acidic environment promotes hydrogen‐bonding interactions, assembling an in‐situ photoreactive Fe‐haloalkyl complex capable of generating haloalkyl radicals when combined with Selectfluor. This unlocks fluoropolyhaloalkylation of alkenes, incorporating CF_3_, CF_2_X, and other multi‐halogen motifs, by combining commercially available Selectfluor as the fluorine source under mild conditions, and broadens accessible fluorinated drug‐like structures. Spectroscopic studies support the acid‐facilitated LMCT excitation path. The work pioneers the activation of challenging substrates, such as unactivated alkenes, within the realm of iron photocatalysis via LMCT.

**Scheme 58:**
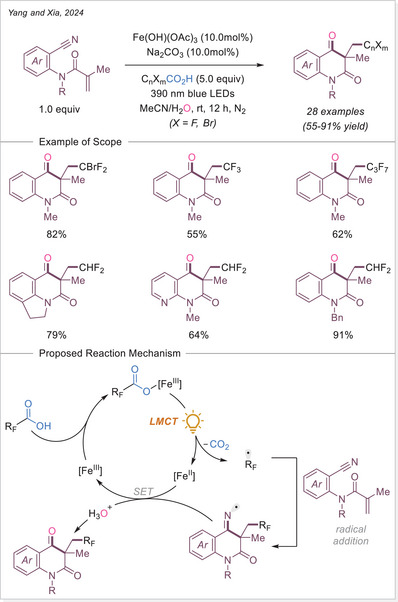
Iron‐photocatalyzed decarboxylative cascade fluoroalkylation of alkenes.

Xia and co investigators also reported in the same year a cascade cyclization approach for synthesizing fluoroalkylated quinolin‐2,4‐diones. Using Fe(OH)(OAc)_2_ under visible light, fluoroalkyl carboxylic acids undergo LMCT mediated decarboxylation to give radicals that trigger intra‐molecular addition onto *N*‐(2‐cyanophenyl)‐*N*‐methylacrylamides, forming cyclized products in 55–91% yields (Scheme 58).^[^ ^120^ ^]^ The methodology achieves broad fluoroalkylation (CF_3_, CF_2_H, etc.) and demonstrates scalability up to gram‐scale in continuous flow. Mechanistic evidence supports radical initiation via LMCT and intramolecular radical addition/cyclization. This work merges LMCT radical generation with structure‐directing cascade chemistry, offering streamlined access to medicinally relevant fluorinated heterocycles.

Very recent work by Fang and co‐workers established an iron‐photocatalyzed decarboxylative cascade alkylarylation of *N*‐arylacrylamides with carboxylic acids to access 3,3‐disubstituted oxindoles under visible light.^[^ ^121^ ^]^ This method shows broad substrate scope and several of accessed oxindole displayed an anti‐inflammatory activity through nitric oxide suppression.

##### Addition to the C = N bonds

**Scheme 59:**
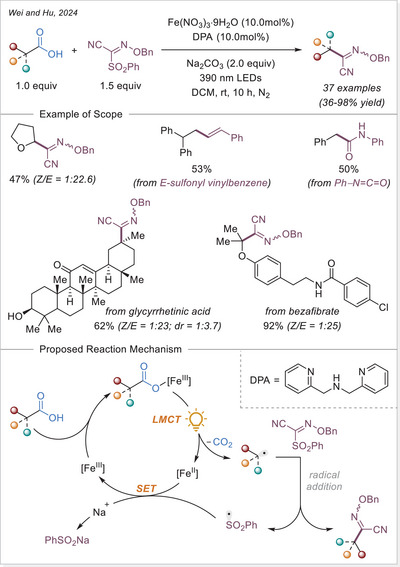
Radical‐mediated decarboxylative C − C Coupling of carboxylic acids via iron photocatalysis. DPA, di(2‐picolyl)amine.

In 2024, Wei and Hu group introduced a broad‐spectrum iron‐photocatalyzed decarboxylative protocol enabling both C─C (Scheme 59) and C─S bond (Scheme 66) formations from aliphatic carboxylic acids.^[^ ^122^ ^]^ This protocol showcased an efficient generation of alkyl radical via LMCT, which then added selectively to a diverse array of radical acceptors including sulfonyl oximes, sulfonyl alkenes, sulfonyl alkynes, isocyanates, and S‐phenyl benzenethiosulphonate precursors using Fe(NO_3_)_3_•9H_2_O as catalyst. This method yielded oxime ether, alkenylated, alkynylated, amide, and thioether products over 35 examples, often with excellent yields up to 98%. Mechanistic studies support a unified radical generation process via a photoinduced LMCT process.

**Scheme 60:**
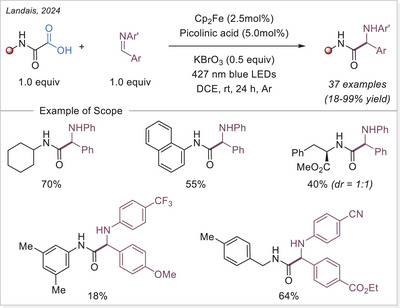
Visible light‐mediated iron‐catalyzed addition of carbamoyl radicals to imines. Cp, cyclopentadienyl.

In 2024, Landais and coworkers demonstrated that oxamic acids undergo LMCT‐driven decarboxylation under visible light using ferrocene as photocatalyst/Lewis acid, generating carbamoyl radicals that add to imines (Scheme 60).^[^ ^123^ ^]^ This transformation delivers α‐amino acid amides in good yields and can be executed either with pre‐formed imines or via a one‐pot three‐component process (aldehyde, amine, oxamic acid). The methodology tolerates a wide range of functional groups (e.g., halogens, esters, nitriles), operates under mild conditions, and is scalable. Mechanistic studies, including TEMPO trapping and radical trapping with olefins, support the formation of carbamoyl radicals by LMCT. This work highlights the strategic use of iron photocatalysis via LMCT for the sustainable synthesis of amino acid derivatives.

**Scheme 61:**
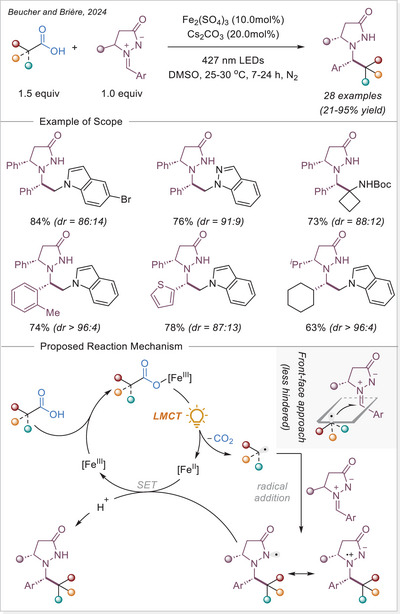
Iron‐catalyzed decarboxylative radical addition to chiral azomethine imines.

Brière and team in the same year reported an iron‐catalyzed, redox‐neutral decarboxylative addition onto chiral azomethine imines under blue‐light irradiation (427 nm). Fe(III)–carboxylate undergoes LMCT/homolysis/decarboxylation sequence to generate alkyl radicals that add to azomethine imines, furnishing cyclic hydrazine derivatives with high diastereoselectivity (dr = 82:18 to > 96:4) (Scheme 61).^[^ ^124^ ^]^ The ligand‐free system uses earth‐abundant iron and proceeds at room temperature with broad substrate scope. This methodology enables access to chiral heterocycles under mild conditions, showcasing LMCT's capacity in enantioselective radical synthesis.

##### Other‐type reactions

**Scheme 62:**
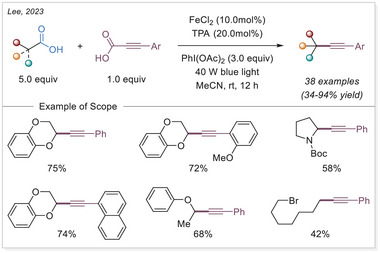
Iron‐photocatalyzed double decarboxylative coupling reactions of alkynoic acids and alkyl carboxylic acids. TPA, (2‐pyridylmethyl)amine.

In 2023, Lee group reported an Fe(III)‐catalyzed visible‐light‐induced strategy for constructing C(sp)─C(sp^3^) bonds by coupling terminal alkynoic acids and aliphatic carboxylic acids via double decarboxylation (Scheme 62).^[^ ^125^ ^]^ The reaction proceeds through sequential LMCT processes that independently generate both alkynyl and alkyl radicals, which then couple to form alkyl‐substituted alkynes in good yields. This method tolerates a broad range of functional groups and avoids the use of external oxidants, ligands, or photocatalysts. The study highlights a rare and efficient decarboxylative alkylation of alkynoic acids and expands the synthetic utility of iron‐photocatalyzed radical chemistry for accessing complex alkyne derivatives under mild conditions.

**Scheme 63:**
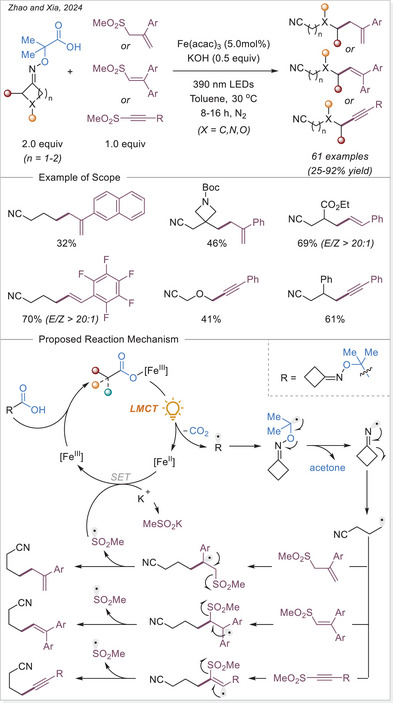
Iron‐catalyzed decarboxylative fragmentation‐alkynylation, ‐alkenylation, and ‐alkylation cascade.

Zhao, and Xia group reported a versatile iron‐catalyzed, visible‐light‐driven radical cascade that combines decarboxylative fragmentation with diverse C─C bond‐forming reactions (Scheme 63).^[^ ^126^ ^]^ The process begins with LMCT activation of aliphatic carboxylates using Fe(acac)_3_ under blue light, generating alkyl radicals that undergo β‐scission to form secondary or stabilized radicals and nitrile functionality. These radicals are then trapped by electrophilic partners such as alkynyl bromides, α,β‐unsaturated ketones, or alkyl halides, enabling efficient synthesis of alkynes, alkenes, and saturated hydrocarbons, respectively. The reaction proceeds under external ligand‐free conditions, is compatible with a wide range of functional groups, and allows for modular construction of complex molecules. Mechanistic studies, including radical clock experiments and control reactions, support a photoinduced LMCT pathway followed by radical relay. This work demonstrates the strategic potential of iron photocatalysis in building molecular complexity via cascade radical transformations.

**Scheme 64:**
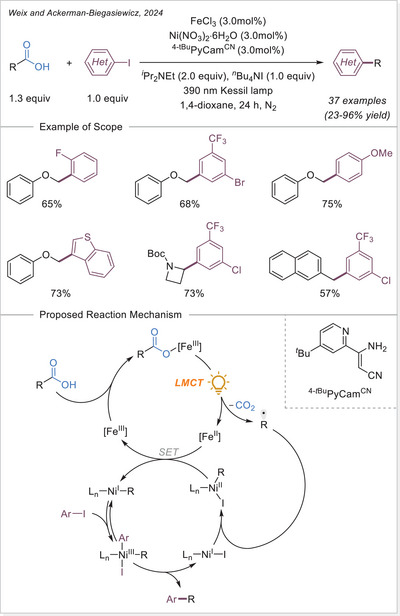
Decarboxylative cross‐coupling enabled by Fe and Ni metallaphotoredox catalysis.

In 2024, Weix and Ackerman‐Biegasiewicz group presents a dual catalytic system that merges iron‐based LMCT photocatalysis with nickel catalysis to enable decarboxylative cross‐coupling between alkyl carboxylic acids and aryl halides (Scheme 64).^[^ ^127^ ^]^ Under visible‐light irradiation, Fe(III) salts generate alkyl radicals from carboxylates via LMCT, which are then intercepted by Ni(I)─aryl intermediates to forge C(sp^3^)─C(sp^2^) bonds. This method accommodates a wide array of primary, secondary, and tertiary carboxylic acids, as well as various aryl bromides and iodides, offering a modular and sustainable route to alkylated arenes. The process operates under mild conditions demonstrating the synergistic potential of dual iron/nickel catalysis for cross‐coupling via radical pathways.

#### 4.2.2 Decarboxylative C─N Bond Formation

**Scheme 65:**
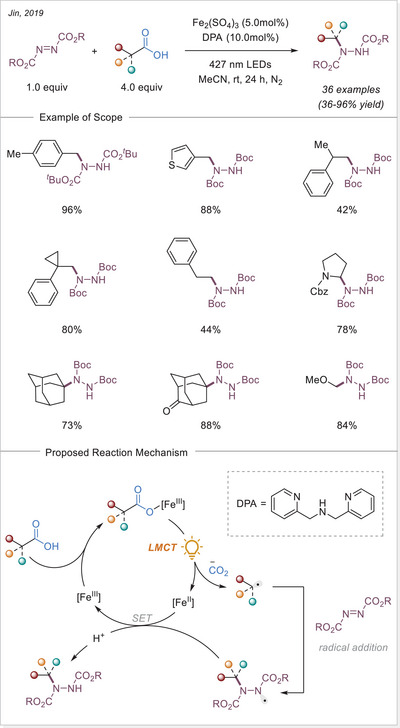
Fe‐catalyzed decarboxylative amidation. DPA, di(2‐picolyl)amine.

The field of iron‐mediated LMCT‐driven decarboxylative C─N bond formation has evolved significantly in recent years. In 2019, Jin and coworkers developed a ligand‐accelerated iron(III) photocatalytic system for decarboxylative radical generation under visible light. Using inexpensive Fe_2_(SO_4_)_3_ and di‐(2‐picolyl)amine (DPA) as a chelating ligand, aliphatic carboxylic acids undergo LMCT to release alkyl radicals (Scheme 65).^[^ ^23^ ^]^

These radicals participate in two distinct types of bond‐forming processes: C─C bond formation and C─N bond formation via radical trapping by azodicarboxylates. The reaction proceeds under blue LED irradiation at room temperature without requiring an external photocatalyst or oxidant. Mechanistic studies, including radical trapping and control experiments, support the involvement of photoinduced LMCT as the key activation mode, where the Fe(III)─carboxylate complex absorbs visible light, undergoes homolysis, and generates alkyl radicals. The addition of the DPA ligand significantly enhances reactivity, likely by stabilizing the Fe(III) center.

**Scheme 66:**
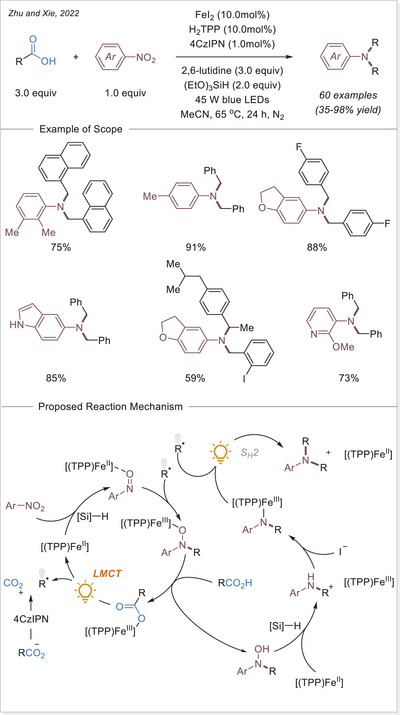
Fe‐catalyzed decarboxylative tandem C─N coupling with nitroarenes. H_2_TPP, tetraphenylporphyrin.

In 2022, Zhu and Xie reported a visible‐light‐driven tandem decarboxylative C─N coupling employing FeI_2_ and 1,2,3,5‐tetrakis(carbazol‐9‐yl)‐4,6‐dicyanobenzene (4CzIPN) photocatalyst to convert carboxylic acids and nitroarenes into aromatic tertiary amines in high yields (35–98%; Scheme 66).^[^ ^128^ ^]^ The reaction shows broad functional group tolerance, including ethers, halogens, esters, ketones, amides, heterocycles, and even allows unsymmetrical tertiary amine synthesis using two different acids. Mechanistic studies (including TEMPO trapping, crossover experiments, and UVVis analysis) indicate that LMCTinduced benzyl radical formation and an arylamine─Fe(III) intermediate undergo a bimolecular homolytic substitution (S_H_2) to form the C─N bond. Notably, NaI plays a key role in facilitating the formation of the Fe‐amine intermediate, and the pathway avoids overalkylation typical of classic S_N_2 routes. This method delivers tertiary amines under mild reaction conditions, showcasing a novel S_H_2 mechanism in iron‐catalyzed radical amination.

**Scheme 67:**
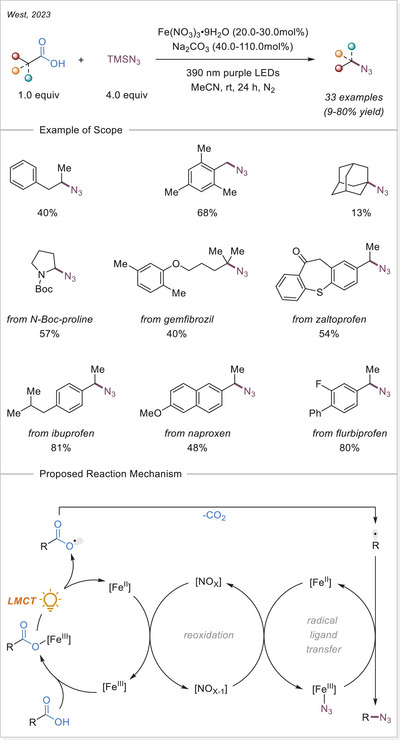
Photochemical iron‐catalyzed decarboxylative azidation.

In 2023, West group demonstrated a visible‐light‐driven, iron‐catalyzed decarboxylative azidation using inexpensive Fe(NO_3_)_3_ **·**9H_2_O and TMSN_3_, enabling direct conversion of both activated and unactivated carboxylic acids into alkyl azides without any external oxidant (Scheme 67).^[^ ^129^ ^]^ The protocol tolerates a broad substrate scope, including primary, secondary, and tertiary acids, and is compatible with functional groups such as esters, amides, heterocycles, and complex drug‐like motifs. Mechanistic studies support a two‑step radical pathway: LMCT from the photoexcited Fe(III)–carboxylate generates alkyl radicals, which then capture azide via radical ligand transfer (RLT) from Fe(III)–N_3_ complexes. The nitrate counterion functions both as ligand and internal oxidant, reoxidizing Fe(II) back to Fe(III) and thereby enabling catalytic turnover in the absence of external oxidants.

**Scheme 68:**
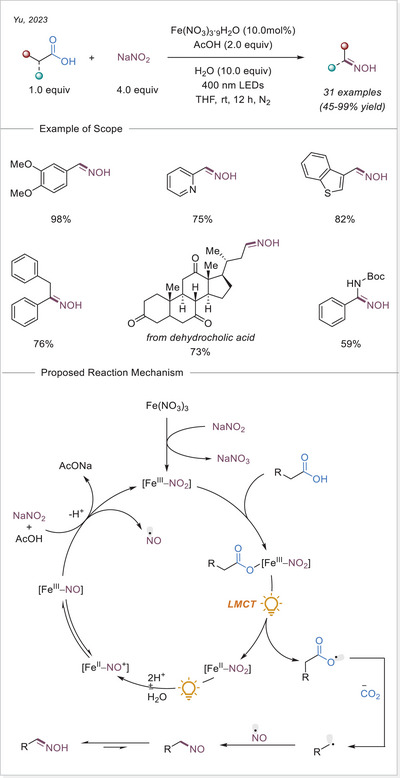
Iron‐catalyzed decarboxylative C − N coupling reaction of alkyl carboxylic acids with NaNO_2_.

In the same year, Yu and coworkers report a visible‐light‐driven iron‐catalyzed decarboxylative C─N coupling of aliphatic carboxylic acids with sodium nitrite, efficiently generating oximes under mild conditions (Scheme 68).^[^ ^130^ ^]^ Utilizing simple Fe salts and blue LEDs, the method enables broad substrate scope, including primary, secondary, and tertiary acids, with good functional‐group tolerance. Mechanistic data support LMCT‐induced alkyl radical generation from Fe‐carboxylate complexes, followed by radical addition to nitric oxide (•NO) radical derived from nitrite.

**Scheme 69:**
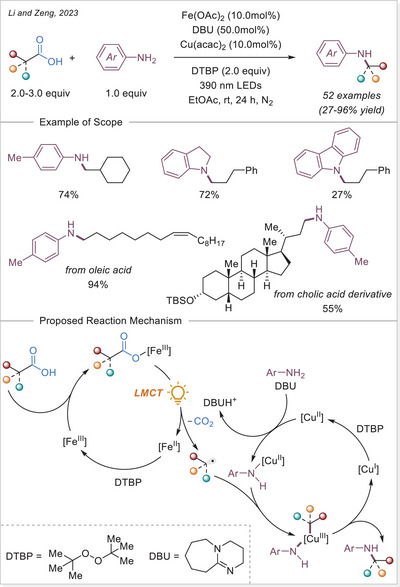
Merging photoinduced iron‐catalyzed decarboxylation with copper catalysis for C − N coupling. acac, acetylacetonate.

Zeng and coworkers advanced the field by integrating iron with copper catalysis. In their dual catalytic platform, alkyl radicals derived from Fe(III)‐mediated LMCT were intercepted by a copper catalyst to forge C─N bond, expanding substrate flexibility and reaction scope (Scheme 69).^[^ ^113^ ^]^ This modular design enabled selective coupling with a variety of nitrogen nucleophiles and provided valuable mechanistic insight into radical relay strategies involving iron and transition metal partners. Collectively, these contributions underscore the growing utility of iron photocatalysis for sustainable radical‐based C─N bond formation, highlighting its broad substrate scope, functional group tolerance, and compatibility with both stoichiometric and dual catalytic systems.

#### 4.2.3 Decarboxylative C─Chalcogen Bond Formation

**Scheme 70:**
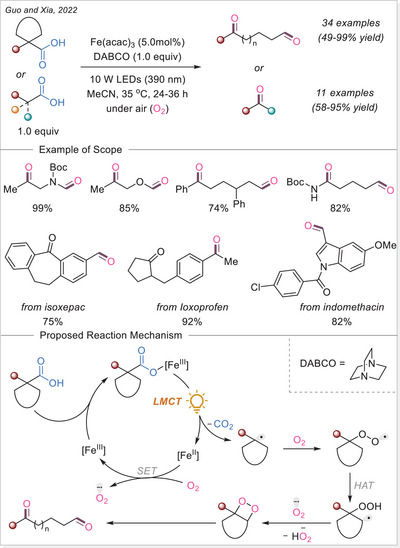
Iron‐catalyzed decarboxylative oxygenation of aliphatic carboxylic acids. Boc = ‐C(O)O*^t^* Bu.

In 2022, Guo and Xia introduced an iron‐photocatalyzed LMCT‐driven ring‐opening strategy of cyclic tertiary carboxylic acids (Scheme 70).^[^ ^131^ ^]^ Using Fe(acac)_3_ under blue LED irradiation, they achieved homolytic C─C bond cleavage to generate tertiary alkyl radicals, which underwent oxygen‐trapping to result dicarbonyl compounds in moderate to high yields. The reaction proceeded under mild conditions, tolerating various functional groups, such as ketones, esters, and Boc‐protected amines. This study emphasized the ability of iron‐photocatalyzed LMCT‐mediated C─O bond formation to access highly functionalized product from simple cyclic carboxylic acids.

**Scheme 71:**
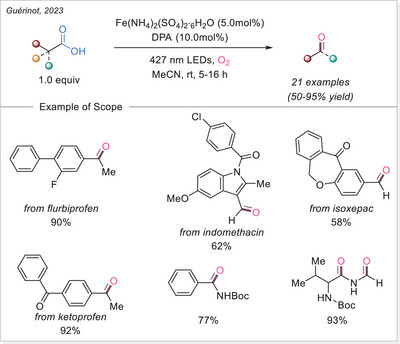
Iron‐catalyzed, light‐driven decarboxylative oxygenation.

In 2023, Guérinot and coworkers described a decarboxylative oxygenation of aliphatic carboxylic acids under iron photocatalysis (Scheme 71).^[^ ^132^ ^]^ Their mild reaction conditions utilized molecular oxygen to convert the carboxylic acids into ketones, aldehydes, and amides. The system accommodates both aliphatic and aromatic substrates, employing dioxygen as a green oxidant, and demonstrates high functional group tolerance along with operational simplicity. Mechanistic investigation, in particular, UV‐Vis spectroscopic studies, confirmed the formation of photoactive iron–carboxylate complexes, supporting the LMCT‐induced radical generation pathway. Spectral changes upon light irradiation indicated the consumption of these complexes, consistent with the LMCT‐mediated decarboxylation mechanism.

**Scheme 72:**
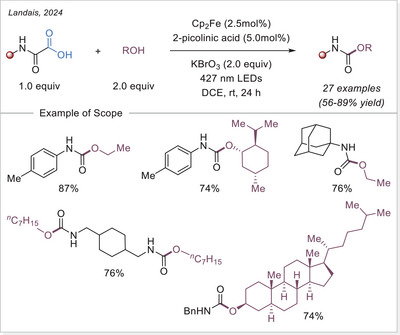
Iron‐catalyzed oxidative decarboxylation of oxamic acids.

Subsequently, Landais and coworkers reported a rapid photochemical decarboxylative oxidation of oxamic acids using ferrocene as a photocatalyst, 2‐picolinic acid as a ligand, and potassium bromate as an oxidant (Scheme 72).^[^ ^133^ ^]^ They achieved oxidative cleavage leading to carbamates or urethanes in good yields (56–89%). This one‐pot method avoids the direct utilization of isocyanates, improving safety and efficiency. Mechanistic studies support a decarboxylation pathway involving carbamoyl radical intermediates that rearranges to isocyanates before being trapped by alcohols. The method provides an efficient route to urethanes from stable and easily handled precursors.

**Scheme 73:**
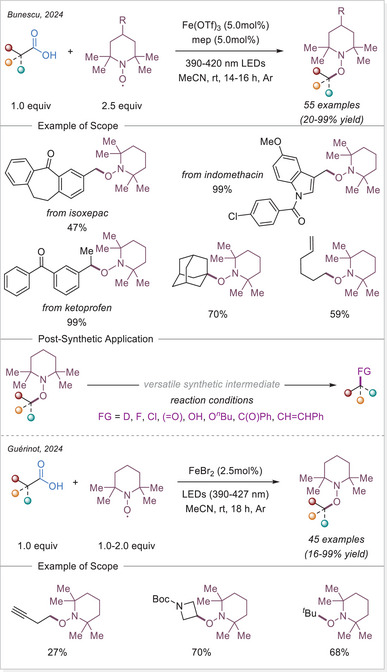
Iron‐catalyzed decarboxylative oxygenation of aliphatic carboxylic acids and their synthetic applications.

In 2024, the Bunescu group developed a mild, visible‐light‐induced, iron‐catalyzed strategy which enables broad and efficient decarboxylative C(sp^3^)–O oxygenation of diverse aliphatic carboxylic acids using TEMPO derivatives (Scheme 73, top).^[^ ^55^ ^]^ The method tolerates a wide range of substrates including sterically and electronically diverse acids and has been successfully applied to late‐stage oxygenation of biologically active molecules. Mechanistic studies (kinetic analysis, EPR, UV‐Vis, electrochemistry, HRMS, and DFT) reveal that photoinduced‐LMCT in the Fe–O_2_CR complex generates alkyl radicals, which are intercepted by TEMPO radical. Importantly, TEMPO acts in a triple role as oxygenation reagent, internal oxidant for Fe(II) to Fe(III) turnover, and internal base for deprotonation. The resulting TEMPO‐adducts act as versatile synthetic intermediates for diverse post‐functionalization reactions, enabling the formation of both C─C and C─heteroatom bonds. These transformations are efficiently carried out using commercial organophotocatalysts with various nucleophiles. In 2024, at the same time, Guérinot group also disclosed a similar approach of transforming carboxylic acids into alkoxyamines utilizing FeBr_2_ in combination with TEMPO under the visible‐light irradiation strategy (Scheme 73, bottom).^[^ ^56^ ^]^

In 2020, Lei and Jin developed an iron‐catalyzed photochemical method for intramolecular C─H oxygenation of 2‐biphenylcarboxylic acids under mild conditions.^[^ ^134^ ^]^ This strategy accommodated various substituents on both aryl rings, delivering oxygenated products in high yields. Unlike typical radical pathways, this transformation proceeded without decarboxylation, likely involving aryl carboxylate–iron(III) complexes generating aroyloxy radicals under visible light.

**Scheme 74:**
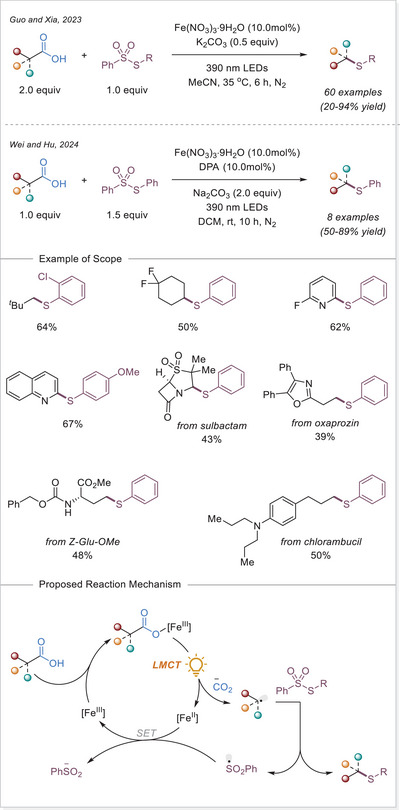
Iron‐catalyzed C─S bond‐forming reaction of carboxylic acids.

In 2023, Xia and coworkers developed a robust iron‐catalyzed protocol in which alkyl carboxylic acids, including the heteroaryl‐tethered, are transformed into thioethers by coupling with S‐aryl benzenethiosulphonate precursors via photoinduced LMCT from Fe‐carboxylate complexes (Scheme 74).^[^ ^135^ ^]^ Remarkably, this one‐step process tolerates a wide array of functional groups. Interestingly, this method also directly affords the corresponding sulfoxide products under aerobic reaction conditions, showcasing its versatility. Operational simplicity and scalability, combined with the use of inexpensive iron salts, emphasize the sustainability and synthetic value of this approach to access high‐value sulfur‐containing molecules and suggest that LMCT can also utilized for C─S bond formation.

**Scheme 75:**
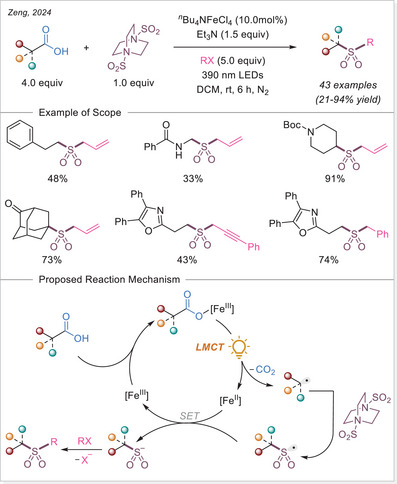
Decarboxylative sulfonylation of carboxylic acids under photomediated iron catalysis.

In 2024, the Zeng group advanced a photoinduced iron‐catalyzed decarboxylative sulfonylation method that transforms a broad range of aliphatic carboxylic acids into sulfones using 1,4‐diazabicyclo[2.2.2]octane•(SO_2_)_2_ (DABSO) as a sulfonylating reagent (Scheme 75).^[^ ^136^ ^]^ The reaction employs inexpensive *^n^* Bu_4_NFeCl_4_ catalyst under mild conditions and tolerates diverse functional groups such as esters, ethers, halides, and heterocycles. Mechanistically, decarboxylation of Fe‐carboxylate complexes occurs via LMCT, generating alkyl radicals that couple with sulfonyl radicals to form the C─S bond, followed by single‐electron reduction and nucleophilic capture steps. The protocol delivers sulfones in moderate to good yields.

#### 4.2.4 Decarboxylative C─Halogen Bond Formation

**Scheme 76:**
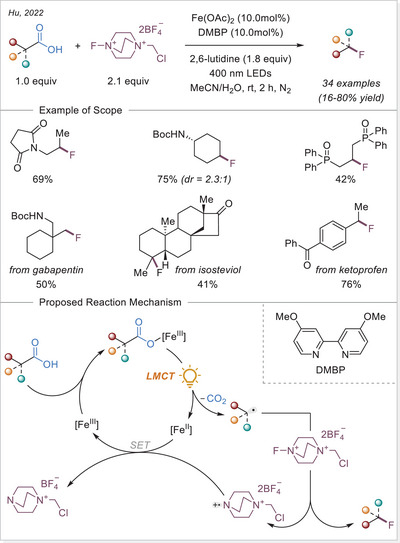
Visible‐light‐induced iron‐catalyzed decarboxylative fluorination of aliphatic carboxylic acids.

In 2022, Hu and coworkers introduced the first iron‐catalyzed photochemical decarboxylative fluorination of aliphatic carboxylic acids, achieving traditionally challenging motifs, alkyl fluorides, under blue LED irradiation (Scheme 76).^[^ ^137^ ^]^ This transformation employs electrophilic fluorine source Selectfluor which also act as oxidant for Fe(II) to Fe(III) transformation. The method accommodates a broad substrate scope (primary to tertiary carboxylic acids), good functional group tolerance, and high selectivity, using inexpensive iron salt and proceeds under mild conditions. This work fills a crucial gap in LMCT‐based decarboxylative functionalization by enabling the access to biologically relevant C─F bond‐containing molecules.

**Scheme 77:**
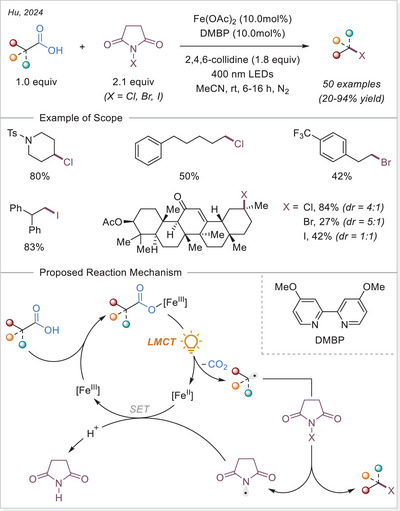
Visible‐light‐induced iron‐catalyzed decarboxylative halogenation of aliphatic carboxylic acids.

In 2024, the same group reported a visible light‐driven iron‐catalyzed decarboxylative halogenation protocol using iron salts under mild conditions, achieving chlorination, bromination, and iodination on aliphatic carboxylic acids (Scheme 77).^[^ ^138^ ^]^ The method tolerates a broad range of functional groups, including benzylic and allylic C(sp^3^)─H bonds, and enables late‐stage functionalization of complex natural products. Mechanistically, Fe(III)‐carboxylate will undergo a LMCT/homolysis/carbon dioxide extrusion sequence to generate the alkyl radical which then reacts with *N*‐halosuccinimides to deliver the desired halogenated products. This decarboxylative halogenation process enables a facile halogen incorporation useful for downstream derivatization of carboxylic acids.

Very recently, Jin group introduced an iron‐photocatalyzed decarboxylative bromination of (hetero)aryl carboxylic acids via a ligand‐to‐metal charge‐transfer (LMCT) pathway.^[^ ^139^ ^]^ This protocol employs NaBrO_3_ as both oxidant and bromine source, enabling efficient access to bromoarenes from readily available substrates.

#### 4.2.5 Decarboxylative Protonation/Deuteration and Decarboxyolefination

**Scheme 78:**
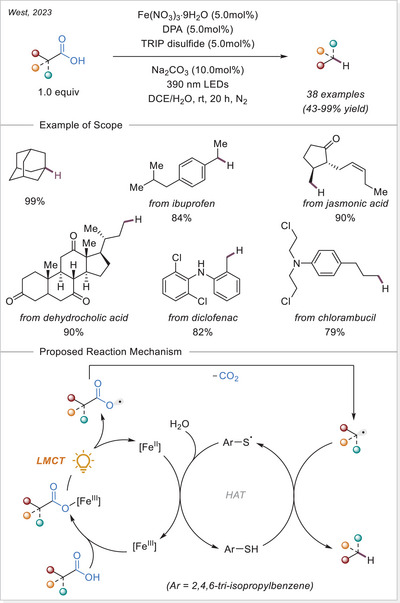
Visible‐light‐induced iron‐catalyzed chemoselective decarboxylative protonation. DPA, di(2‐picolyl)amine; TRIP, 2,4,6‐triisopropylphenyl.

In 2023, West group developed a cooperative iron‐catalyzed, visible‐light‐driven decarboxylative protonation protocol selectively converts carboxylic acids into alkanes under mild conditions, using LMCT‐activated Fe–carboxylate complexes and a proton donor (Scheme 78).^[^ ^140^ ^]^ HAT is favored, producing alkanes with high chemoselectivity, avoiding side reactions. The reaction proceeds under oxidant‐free conditions, tolerating diverse functional groups. Mechanistic insights support LMCT‐mediated radical formation followed by HAT to form the protonated products.

**Scheme 79:**
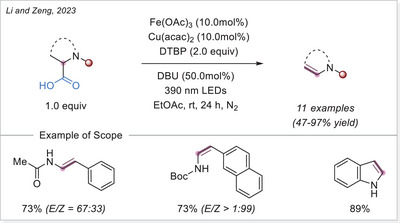
Visible‐light‐induced iron‐catalyzed decarboxyolefination.

Zeng and Li reported the dual iron/copper photoredox system in which aliphatic carboxylic acids undergo decarboxylative olefination of amino acids, forming internal alkenes (C═C bonds) in high regioselectivity (Scheme 79).^[^ ^113^ ^]^ Instead of forming amination products, these substrates undergo β‐H elimination to produce alkenes, likely due to copper intermediate stabilization by adjacent nitrogen atoms. Substrate encompassing with protecting groups (e.g., Boc versus acetyl) significantly influence *Z*/*E*‐selectivity.

### 4.3 LMCT of Alkoxy‐Iron Species in Organic Synthesis

The photoinduced homolytic cleavage of Fe‐alkoxy bonds via LMCT generates highly reactive alkoxyl radicals under mild photochemical conditions. Upon light excitation, electron transfer from the alkoxide ligand to the Fe(III)‐center induces homolytic bond scission and the formation of reduced Fe(II). The resulting alkoxyl radicals can undergo 1,5‐HAT, β‐scission, or direct functionalization reactions. This strategy provides a versatile platform for remote C─H activation, oxidative rearrangements, and deconstructive functionalization of alcohol derivatives. Recent advances highlight the potential of activation of Fe–alkoxide complexes via LMCT as a sustainable and tunable method for radical‐mediated transformations.^[^ ^141^ ^]^

**Scheme 80:**
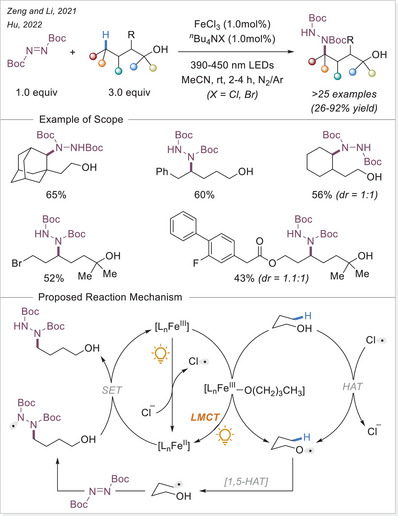
Iron‐catalyzed remote δ C(sp^3^)−H amination of free alcohol.

In 2021, Zeng and Li reported the FeCl_3_‐catalyzed C(sp^3^)−H amination of aliphatic free alcohol under 390 nm light irradiation (Scheme 80).^[^ ^142^ ^]^ This reaction directly converts the readily available free alcohol into δ‐amino alcohols under oxidant‐free mild reaction conditions. A similar transformation was also reported by the Hu group under 450 nm LED irradiation.^[^ ^143^ ^]^ Both studies proposed a comparable mechanism, in which alkoxy radicals were generated either by LMCT of Fe–OR species or by chloride radical‐mediated hydrogen abstraction from free alcohol. Subsequently, an intramolecular 1,5‐HAT generated an alkyl radical, which then underwent addition to diazenes, affording the desired product. They demonstrated the utility of this method by highlighting its broad substrate scope and potential for diverse synthetic applications, making it a highly attractive approach for the efficient synthesis of δ‐functionalized alcohols.

**Scheme 81:**
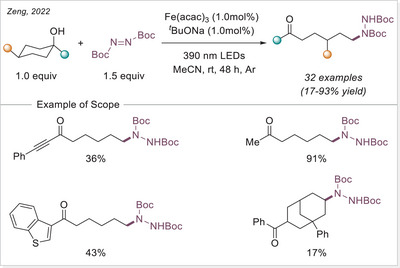
Fe–alkoxide LMCT mediated C − C bond cleavage and amination of unstrained cyclic alcohols.

Building on their previous work in 2022, the Zeng group later developed a chlorine‐free catalytic cycle for the iron‐catalyzed C─C bond cleavage and amination of cyclic alcohols (Scheme 81).^[^ ^144^ ^]^ Using 2.0mol% Fe(acac)_3_ and 6.0mol% *^t^* BuONa under 390 nm LED irradiation, this strategy facilitated the efficient conversion of cyclic α‐substituted, strained tertiary and secondary alcohols with different ring sizes into amino carbonyl compounds with moderate to excellent yields. The key step involved LMCT‐induced homolytic cleavage of the Fe‐OR bond, generating an alkoxy radical that underwent β‐scission to form an alkyl radical, which was subsequently trapped by di‐*tert*‐butyl azodicarboxylate (DBAD) to furnish the final product.

**Scheme 82:**
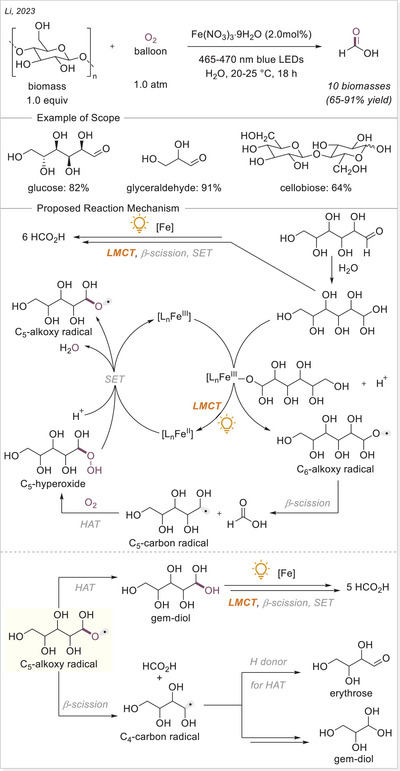
Fe‐alkoxide LMCT‐mediated transformation of biomass into formic acid.

Following this strategy, Li and coworkers employed Fe(NO_3_)_3_•9H_2_O as a catalyst to achieve the visible‐light‐driven conversion of biomass‐derived different polyhydric alcohols into formic acid (Scheme 82).^[^ ^145^ ^]^ The reaction proceeds in an aqueous medium at ambient temperature, reaching yields of up to 91%. This transformation also follows an LMCT‐mediated alkoxy radical formation, followed by β‐scission, HAT and oxygen trapping, showcasing the potential of iron‐based LMCT catalysis in sustainable biomass vaporization.

**Scheme 83:**
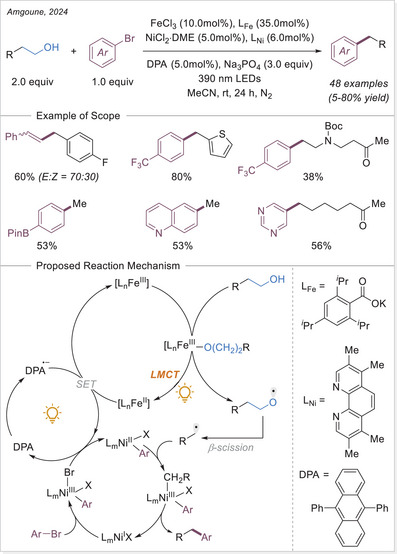
Photocatalytic cross‐coupling of alcohols with aryl halides enabled by synergistic nickel and iron catalysis.

In 2024, Amgoune and Tran reported a versatile photo‐dual catalytic strategy employing Fe/Ni catalysis to facilitate a broad range of cross‐coupling reactions using both primary and tertiary alcohols (Scheme 83).^[^ ^146^ ^]^ The system employs a photoactive Fe‐LMCT catalyst to generate alkoxy radicals from a wide range of alcohols, while a Ni catalyst facilitates oxidative addition of the aryl halide, radical capture, and subsequent C─C bond formation. Catalytic turnover is achieved through an organophotoredox catalyst functioning as a redox mediator between the two catalytic cycles. Mechanistic investigations have confirmed the distinct roles of each catalytic component and highlighted the critical influence of the ancillary ligand environment on the iron catalyst, which is essential for achieving broad substrate scope and high efficiency. Their substrate scope encompasses (i) dehydroxymethylative arylation of aliphatic alcohols using broad tolerance to electronic properties of aryl bromide and primary alcohol, (ii) remote arylation of hindered cyclic alcohols to afford arylated ketones, and (iii) methylation of aryl halides using tertiary alcohols as a methyl radical source.

In the similar time frame, Mei, Zou, and Ma combinedly reported a similar approach of remote arylation by Fe─Ni dual catalysis.^[^ ^147^ ^]^ They harness photoinduced LMCT to generate alkyl radicals from unactivated cyclic alcohols, while these radicals are intercepted in‐situ by catalytic nickel species produced via cathodic reduction, creating Ni–alkyl intermediates that arise from convergent paired electrolysis. Simultaneously, the paired oxidation at the anode regenerates both the active iron photocatalyst and nickel catalyst.

**Scheme 84:**
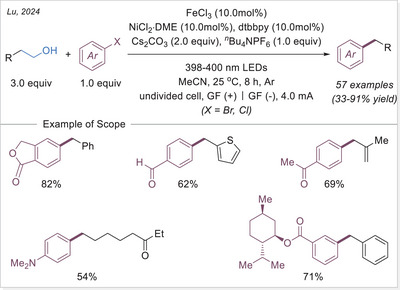
Photoelectrochemical Fe/Ni dual catalyzed C − C functionalization of alcohols. GF, graphite felt; dtbbpy, 4,4‐di‐*tert*‐butyl‐2,2‐dipyridyl; DME, 1,2‐dimethoxyethane.

In the same year, Lu group reported a photoelectrocatalytic strategy using dual Fe/Ni catalysis for C(sp^2^)–C(sp^3^) coupling reactions (Scheme 84).^[^ ^148^ ^]^ The mechanistic pathway involves an initial ligand‐metal charge‐transfer (LMCT) event leading to the homolysis of the Fe–OR bond, followed by β‐cleavage, which subsequently generates a radical that adds to an aryl‐Ni complex. This transformation is enabled by a photoelectrochemical setup in which iron catalysis at the anode promotes alcohol activation, while nickel catalysis at the cathode enables electrophile activation. Remarkably, this system circumvents the need for stoichiometric chemical reductants or oxidants, thus providing a more sustainable alternative to conventional approaches. This methodology demonstrates broad functional group tolerance, successfully synthesizing over 50 structurally diverse molecules. However, certain functional groups such as free alcohols, thiols, and benzoyl chlorides are incompatible under the reaction conditions. Despite these specific limitations, the system maintains considerable versatility, emphasising its potential as a valuable tool in synthetic organic chemistry.

### 4.4 LMCT of Azide‐Iron Species in Organic Synthesis

LMCT‐mediated cleavage of Fe–azide bonds enable the photochemical generation of nitrogen‐centered azidyl radicals from simple azide salts. Upon light‐induced electron transfer from the azide ligand to the Fe(III) center, homolytic bond cleavage occurs, producing highly reactive azidyl species. These intermediates can engage in alkene functionalization. This strategy provides a direct and mild approach to activating azides for radical chemistry. Fe‐azide LMCT systems hold significant potential for site‐selective and nitrogen‐incorporating transformations under sustainable conditions.

**Scheme 85:**
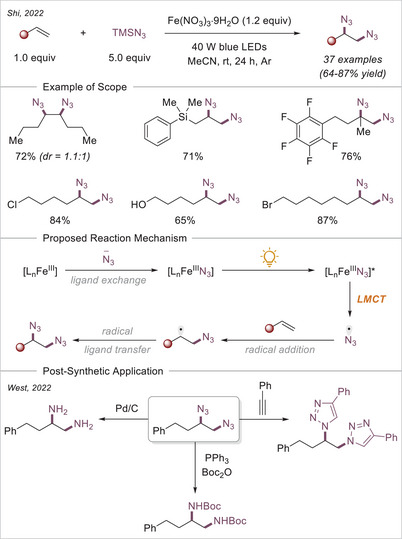
Iron‐mediated LMCT facilitates the 1,2‐diazidation of alkenes.

In 2022, Shi^[^ ^149^ ^]^ and West^[^ ^150^ ^]^ group independently reported a similar approach utilizing LMCT excitation for the 1,2‐diazidation of alkenes using TMSN_3_ (Scheme 85). Their strategy involved a LMCT‐homolysis process, employing an earth‐abundant and environmentally friendly iron salt. This LMCT‐homolysis mechanism facilitates the generation of an electrophilic azidyl radical intermediate from Fe(III)‐N_3_ complexes, which then undergoes radical addition to carbon‐carbon double bonds. The resulting carbon radical intermediate is subsequently intercepted through iron‐mediated azidyl radical ligand transfer (RLT), enabling the formation of the second carbon‐azide bond. This method offers a versatile and chemoselective approach to access structurally diverse diazides from readily available alkenes and unactivated alkenes, without requiring chemical oxidants. Additionally, the West group advanced this reaction by implementing continuous‐flow photolysis, which enhances reaction efficiency through uniform irradiation of the entire reaction mixture. This approach enables shorter reaction times, facilitates large‐scale synthesis, and improves mass balance by minimizing byproduct formation and substrate degradation. They further demonstrated the synthetic utility of the diazide products by subjecting them to click reactions, hydrogenation, and Staudinger reduction, yielding their corresponding products.

**Scheme 86:**
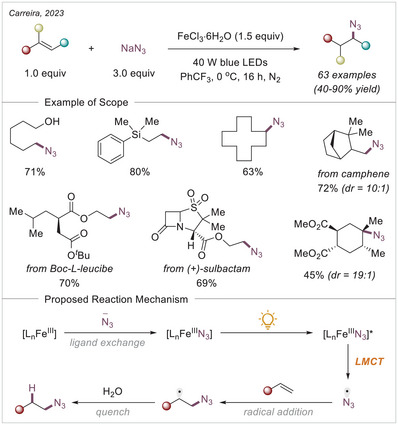
Iron‐mediated photochemical anti‐Markovnikov hydroazidation of unactivated olefins.

In 2023, Carreira and coworkers reported a method for the anti‐Markovnikov hydroazidation of unactivated olefins utilizing bench‐stable NaN_3_ and stoichiometric FeCl_3_•6H_2_O (Scheme 86).^[^ ^151^ ^]^ This transformation enables the efficient synthesis of alkyl azides under mild, ambient conditions and is operationally simple, proceeding in the presence of air and moisture. The reaction demonstrates broad functional group tolerance, making it well‐suited for the late‐stage functionalization of complex molecules. From the mechanistic studies they proposed that N_3_• radical is formed via the LMCT mediated Fe–N_3_ homolysis and water bound to the act as hydrogen atom source in the hydroazidation process.

**Scheme 87:**
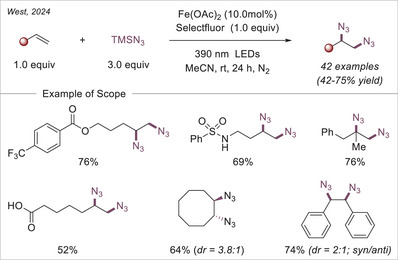
LMCT‐mediated iron‐photocatalyzed diazidation of unactivated alkenes.

In 2024, West group expanded on the previously mentioned LMCT approach for the diazidation of unactivated alkenes, employing a catalytic amount of an iron salt in conjunction with an external oxidant (Scheme 87).^[^ ^152^ ^]^ Preliminary mechanistic studies support the radical nature of these transformations, emphasizing the tandem LMCT/RLT process as a powerful reaction manifold for catalytic olefin difunctionalization.

## 5 Conclusion and Outlook

LMCT excited states in iron complexes have recently emerged as powerful tools for enabling radical‐mediated transformations under visible or near‐UV light. Unlike many other photochemical processes, LMCT involves a pre‐existing ground‐state association between the metal center and the ligand, allowing for direct and efficient bond homolysis upon light excitation, even though the resulting excited states are typically short‐lived. This distinctive reactivity enables the generation of reactive radical species under mild, often room‐temperature conditions, opening new avenues for sustainable and scalable synthetic methodologies. Even though some methods still rely on stoichiometric amounts of Fe(III) complexes, the low cost, commercial availability, and low toxicity of iron help justify their use. Moreover, these systems often provide reactivity profiles that are difficult to replicate with precious metals. The growing diversity in ligand classes and bond homolysis pathways illustrates iron's unique versatility among 3d transition metals.

In this review, we have highlighted major advances in iron‐photoinduced LMCT‐driven radical chemistry for organic synthesis. While halogen radicals have been widely employed in HAT and C─H functionalization reactions, recent studies have broadened the scope of LMCT activation to include the homolysis of carboxylates, alkoxides, and azide. These pathways give access to a variety of radical intermediates including, carboxyl, alkoxyl, and nitrogen‐centered species that enable selective and diverse bond‐forming reactions. From 2020 onward, numerous systems based on Fe(III)─L bonds (L = carboxylate, halide, alkoxide, and azide) have been developed, with applications in C─C and C─X bond formation, HAT, and cross‐coupling reactions. These advances showcase the potential of iron as an earth‐abundant and environmentally benign photocatalyst.

Looking ahead, there is significant potential to expand iron‐photoinduced LMCT catalysis through the design of new ligand environments, deeper insights into structure reactivity relationships, and the development of integrated systems that combine LMCT with dual catalysis or electrochemical activation. Promising directions include asymmetric synthesis, red‐shifted photoactivation, and late‐stage functionalization of complex molecules.

Despite these promising developments, key challenges remain in achieving precise control over reactivity and selectivity. Deeper mechanistic insights into CT dynamics, radical recombination pathways, and structure reactivity relationships through photophysical, electrochemical, and computational studies are essential to guide future catalyst design. Moreover, efforts to expand the substrate scope, particularly in asymmetric transformations, and to reduce reliance on stoichiometric iron reagents, will further enhance the utility of these systems. Looking ahead, the integration of LMCT photocatalysis with dual catalytic approaches, electrochemical activation, and continuous‐flow technologies holds exciting promise for scalable and sustainable synthesis. The development of novel ligand frameworks enabling red‐shifted photoactivation, enhanced selectivity, and even bio‐relevant applications further underscores iron's unique versatility among 3d transition metals. With growing interest from both academia and industry, iron‐based LMCT catalysis is poised to play an increasingly important role in the development of environmentally benign and industrially relevant synthetic methodologies.

## Conflict of Interest

The authors declare no conflict of interest.

## Data Availability

Data sharing is not applicable to this article as no new data were created or analyzed in this study.
